# Redox Oligomer Assembling Hierarchical Reinforced Framework Cathodes for Ultra‐Stable High‐Performance Zinc‐Ion Batteries

**DOI:** 10.1002/advs.202522537

**Published:** 2026-01-04

**Authors:** Shuang Liu, Yiliang Lai, Yinghang Gao, Weihua Tang

**Affiliations:** ^1^ College of Materials College of Chemistry and Chemical Engineering Innovation Laboratory of Energy Materials of Fujian Province (IKKEM) Xiamen University Xiamen Fujian China; ^2^ College of Energy Xiamen University Xiamen Fujian China

**Keywords:** aqueous zinc‐ion battery, energy storage, hierachical nanostructure, organic cathode, reinforced concrete framework

## Abstract

Organic cathodes with maximal redox active‐sites and structural tunability are in great demand for aqueous zinc‐ion batteries (ZIBs). Currently, they are facing challenges of limited capacity, poor cycling stability, and sluggish ion transport in practical applications. In this work, we have developed a universal and efficient strategy to assemble a redox oligomer into a conductive substrate imitating a reinforced concrete framework as freestanding cathodes for high‐performance ultrastable ZIBs. The signature molecule was specifically designed by sandwiching phenoxoline with two benzoquinone units to explore the most redox‐active sites. 2D MXene nanosheets convalently bonded with single‐walled carbon nanotubes to construct hierachical reinforced framework with superior porosity and structural rigidity. Benefiting from superior structural robustness and electrochemical dynamics, the cathodes deliver the highest specific capacity of 339.5 mAh g^−1^ at 0.1 A g^−1^, together with exceptional capacity retention of 87.5% over 65 000 cycles at 10 A g^−1^. The calculated Zn^2+^ diffusion coefficient (10^−8^‐10^−7^ cm^2^ s^−1^) indicates rapid charge transfer kinetics. The assembled pouch cell demonstrates stable power output under various bending angles for a flexible energy supply. This work provides not only a novel nanostructure engineering strategy for fabricating high‐performance hierarchical electrodes but also insights into the design of redox‐rich molecules for next‐generation energy storage.

## Graphical abstract

Redox phenoxoline‐benzoquinone oligomer has been designed as reinforced concrete framework like freestanding cathodes for high‐performance ZIBs. An optimal specific capacity of 339.5 mAh g^−^^1^ was contributed with exceptional retention of 87.5% after 65 000 cycles at 10 A g^−^^1^. Flexible energy storage is also demostrated. The excellent rate performance and cycling stability underscore the electrode design for advanced energy storage.

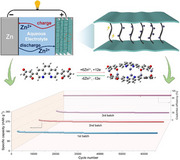

## 1 Introduction

Sustainable human society and environmental development have motivated to explore the utilization and storage of clean energy [1, 2]. Renewable energy sources of solar and wind are limited by uneven resource distribution and high energy extraction costs [3, 4]. It is thus in great demand to develop highly reliable and sustainable electrical energy storage (EES) systems [1, 5]. Lithium‐ion batteries (LIBs) have occupied a significant market share in commercial EES systems over the last few decades due to their high energy density and extended cycle life [6, 7]. However, the widespread usage of LIBs as energy storage systems raises safety and environmental concerns, partly attributed to their manufacturing with hazardous and volatile organic electrolytes, resource‐scarce lithium metal, and vacuum conditions [8, 9, 10]. Recently, aqueous zinc‐ion batteries (ZIBs) have emerged as a viable substitute for LIBs in energy storage systems. Besides cost‐effectiveness and environmental friendliness, ZIBs are advantageous for high theoretical specific capacity (820 mAh g^−1^ and 5855 mAh cm^−3^), low redox potential (−0.76 V versus the standard hydrogen electrode), and abundant zinc metal resources [11, 12, 13]. Till now, large research efforts have been devoted to develop inorganic cathode materials for ZIBs, such as manganese oxides [14], vanadium oxides [15], and Prussian blue analogs [16], Despite high energy density, inorganic electrodes suffer from their inferior rate capability and poor cycling stability caused by structural alterations and active material dissolution to hinder their usage [17, 18]. Thus, it is imperative to explore new cathode materials for ZIBs with excellent performance.

Compared to inorganic materials, organic molecules emerge as promising cathode candidates for ZIBs, due to their advantages of low cost, structural diversity, abundant redox active sites, and design flexibility [19, 20, 21, 22, 23]. Currently, the design of organic cathode materials mainly focuses on organic molecules containing carbonyl and imine functionalities [24, 25]. These n‐type organic compounds can be reduced to negatively charged states by accepting electrons, allowing them to store and release zinc ions during the discharging and charging process [26, 27]. Most organic cathode candidates, especially quinone and imide derivatives, have low molecular weights but abundant electroactive functional groups, providing high theoretical specific capacities. However, their high solubility and low electric conductivity severely reduce their competitive edge [18]. To address these issues, molecular design of conjugated polymers and conjugation extended oligomers has been explored on one hand [28, 29, 30] On the other hand, hierarchical structures by incorporating redox molecules in 2D conducting substrates such as graphene and emerging MXene (Ti_3_C_2_T_x_) bonded with carbon nanotubes, featuring unique metallic conductivity and functionalized surfaces, have thus been designed as potential hybrid electrodes for efficient and stable energy storage in ZIBs [31, 32].

In this work, we report herein a novel acceptor‐donor‐acceptor (A‐D‐A) type redox compound to intercalate into MXene‐bonded single‐walled carbon nanotubes (SWCNTs) nanosheets as free‐standing film cathodes (PTB@MXSC) for ultra‐stable efficient ZIBs. The A‐D‐A molecule entitled 2,2′‐(1,10‐phenanthroline‐3,8‐diyl)bis(cyclohexa‐2,5‐diene‐1,4‐dione) (PTB) contains two structural motifs as electron accepting benzoquinone (BQ) and donating phenoxoline (PA) units, while PTB@MXSC featuring a conductive porous substrate imitating a reinforced concrete framework exempts from usage of activated carbon and binders for conventional cathodes. Morphological and structural characterization indicates that the introduction of SWCNTs broadens the interlayer space of MXene to stabilize the few‐layered nanosheet structure. This ordered layered structure enables the uniform dispersion of PTB particles to take full advantage of active sites for efficient intercalation of Zn^2+^. The optimal PTB@MXSC cathode affords the highest discharge specific capacity of 339.5 mAh g^−1^ at 0.1 A g^−1^, together with a capacity retention of 87.5% at 10 A g^−1^ after long‐term cycling for 65 000 cycles. The ZIBs boast a maximum power density of 72.16 W kg^−1^ and a highest energy density up to 244.63 Wh kg^−1^. Moreover, a flexible PTB@MXSC//Zn pouch cell can be bent at various angles to power LED lights. This work provides a new strategy for preparing high‐performance ZIBs cathodes.

## 2 Results and Discussion

**FIGURE 1:**
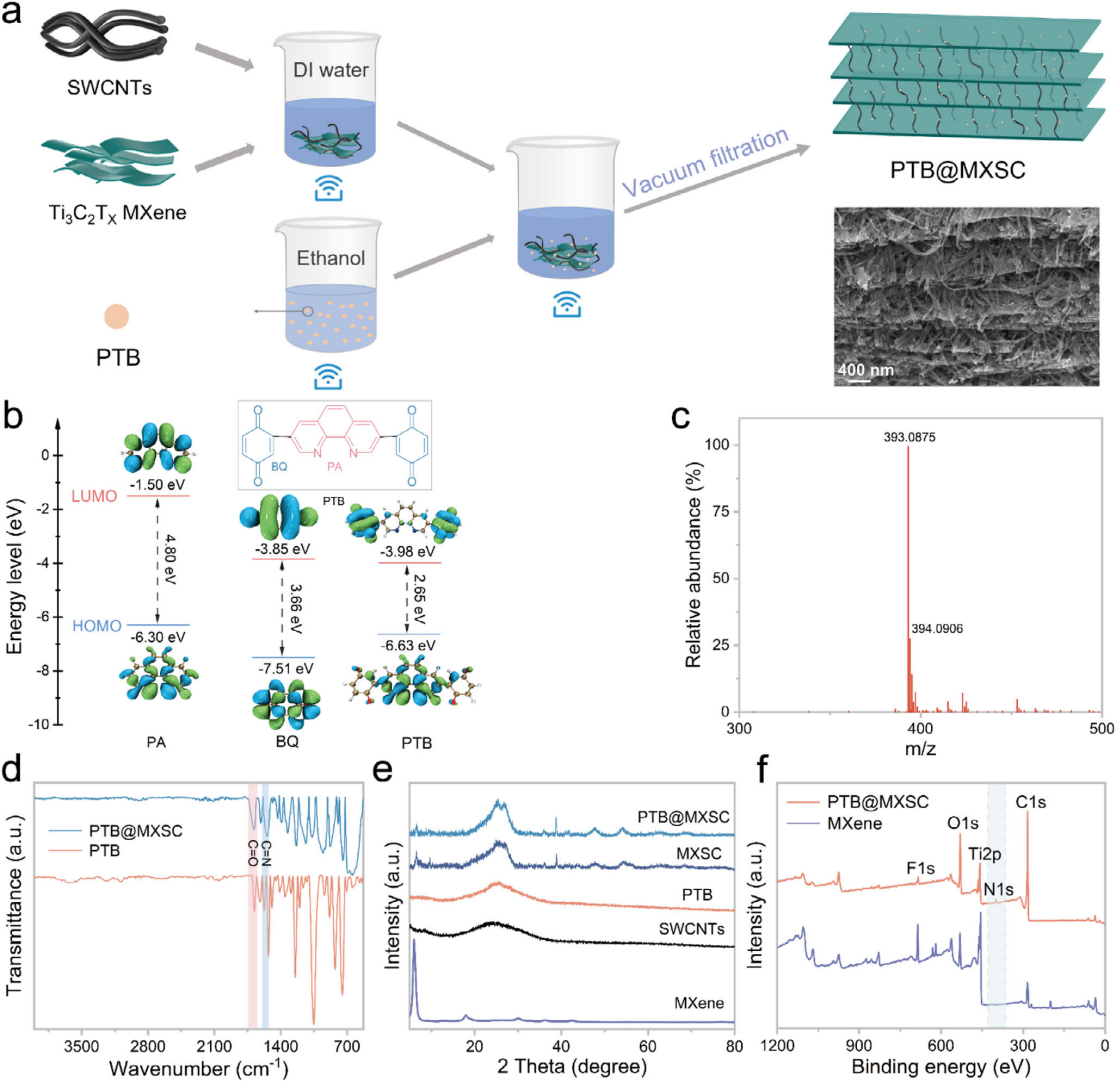
(a) Preparation route of PTB@MXSC. (b) Calculated frontier molecular orbital energy of PA, BQ, and PTB. (c) High resolution mass spectrum of PTB ([M+H]^+^). (d) FTIR spectra of PTB and PTB@MXSC. (e) XRD patterns of MXene, SWCNTs, PTB, MXSC, and PTB@MXSC. (f) XPS spectra of MXene and PTB@MXSC.

PTB was facilely synthesized through a two‐step procedure including a first palladium‐catalysed Suzuki coupling and a followed oxidization of *p*‐dimethoxyphene (Scheme S1 and Figure S1a). Meanwhile, the conductive substrate imitating a reinforced concrete framework was prepared by chemical bonding MXene nanosheets with carboxylated SWCNTs in a water suspension. To this suspension was subsequently added with PTB in designated weight ratios under ultrasonication. The flexible freestanding PTB@MXSC films were further obtained through vacuum filtration (Figure 1a; Figure S1b). To verify the rationality of the molecular design of PTB, the highest occupied molecular orbital (HOMO) and the lowest unoccupied molecular orbital (LUMO) of PTB are calculated for comparing with those of two D/A motifs, i.e., benzoquinone (BQ) and phenoxoline (PA), using density functional theory (DFT) (Figure 1b). PTB showing a downshifted LUMO energy level of −3.98 eV indicates it can achieve an appropriate working potential [33]. PTB exhibits the narrowest HOMO‐LUMO gap, and this bandgap represents the intrinsic electronic conductivity of the molecule [34], which indicates that PTB has a faster electron transport capability. The molecular composition and chemical state of PTB and PTB@MXSC were systematically characterized and verified through a variety of advanced analytical techniques. As shown in Figure 1c, the composition of the compound was confirmed by matrix‐assisted laser desorption/ionization‐time‐of‐flight mass spectrometry (MALDI‐TOF MS). The relative molecular weight determined is 393.0875, matching well with the theoretical molecular weight of PTB.

As Fourier transform infrared (FTIR) spectra illustrated in Figure 1d, PTB exhibits one characteristic peak at 1679 cm^−1^ assigned to the typical C═O stretching vibration and another one at 1575 cm^−1^ attributed to C═N stretching vibration [35, 36]. Such characteristic peaks for C═O and C═N redox‐active sites appear at similar positions in the spectrum of PTB@MXSC. Notably, PTB@MXSC did not show the characteristic peak of −OH groups from MXene in the FTIR spectrum, which can be attributed to the formation of robust covalent linkages between SWCNTs and MXene via dehydration condensation. This reaction was facilitated by the esterification between ‐OH from MXene and carboxyl (−COOH) functionalities from carboxylated SWCNTs, ensuring effective interfacial bonding (Figure S2). The alterations observed in the C 1s, O 1s, and Ti 2p spectra (Figure S3) further demonstrate that MXene and SWCNTs in the PTB@MXSC composite are chemically bonded through C─O─Ti covalent linkages. This specific chemical bonding is responsible for establishing a robust 2D conductive network within the material. To further confirm the successful synthesis of PTB, both ^1^H and ^13^C nuclear magnetic resonance (NMR) spectroscopies were employed (Figure S4). Six peaks assigned for protons from the conjugation skeleton are observed with almost the same integrated area, in good agreement with all hydrogen atoms in different chemical environments in PTB. It should be noted that the relatively low solubility of PTB in DMSO‐d_6_ makes the product peak intensities weaker than those for solvents.

X‐ray diffraction (XRD) was further used for crystalline properties evaluation for PTB@MXSC (Figure 1e). PTB presents an amorphous nature. MXene shows high crystallinity with a sharp, strong diffraction peak observed at 6.1°, The diffraction peak of is observed, corresponding to (002) plane with an average interlayer spacing of 14.47 Å from Bragg's equation. This expanded interlayer spacing can be attributed to the intercalation of water molecules between MXene flakes [37]. The bonding with SWCNTs results in significantly suppressed crystallinity of MXene in MXSC, considering the dramatically reduced content of MXene in MXSC, which contains MXene and SWCNTs in a weight ratio of 7:8. The incorporation of PTB into MXSC further weakened the characteristic peak for (002) plane of MXene. The intensity of the (002) peak is directly related to the degree of ordered stacking of MXene flakes. The increase in the number of MXene layers and the enhancement of structural order lead to higher (002) peak intensity [38], which will be discussed later through electron microscopy analysis of PTB@MXSC. The chemical structure and valence state of elements in freestanding PTB@MXSC film were then investigated via X‐ray photoelectron spectroscopy (XPS). As shown in Figure 1f, MXene exhibits distinct peaks for elements of C, O, F, and Ti, indicating the presence of Ti─C and Ti─O functional groups on MXene [31]. Besides these, PTB@MXSC shows an additional peak for nitrogen (N) in the XPS spectrum to demonstrate the successful intercalation of PTB into the MXSC hierarchical substrate. In one word, the successful synthesis of PTB was verified, together with its distinctive signatures in PTB@MXSC composites clearly identified through detailed structural and spectroscopic analyses.

**FIGURE 2:**
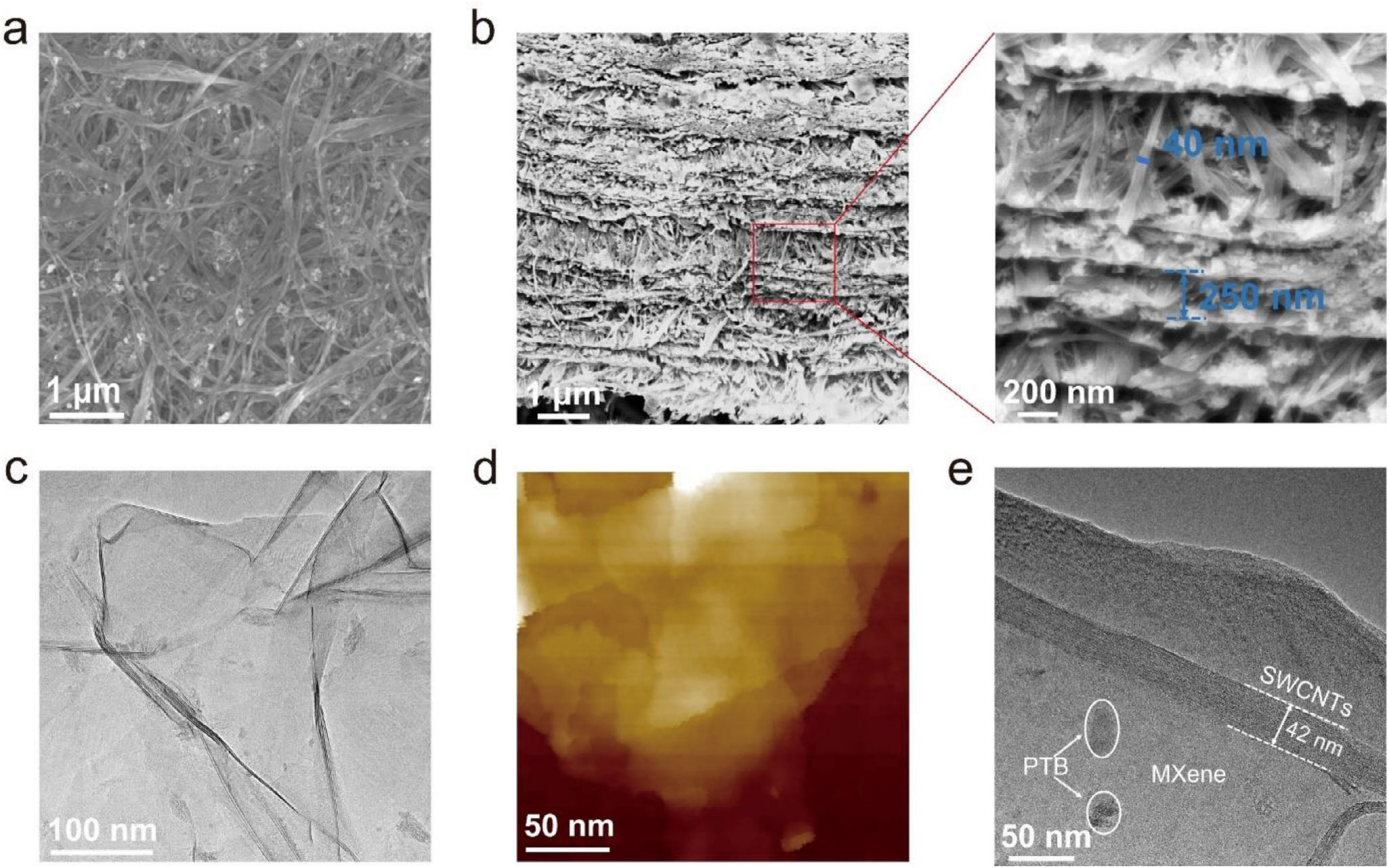
(a) Planar‐view and (b) Cross‐sectional SEM images of the PTB@MXSC. (c) TEM and (d) Atomic force microscope (AFM) images of MXene. (e) TEM image of PTB@MXSC.

Multiple electron microscopy techniques were employed to further explore the structure and morphology evolution of the prepared freestanding film. As revealed in Figure S5, SEM images reveal the nanoscale irregular PTB bulks and bundles of SWCNTs with individual diameters of ∼3 nm self‐assembled through intertubular van der Waals interactions. The planar SEM image of PTB@MXSC composite shows a homogeneous hierarchical nanostructure, where SWCNTs fibers bonded with MXene form highly ordered 3D porous substrates imitating a reinforced concrete framework to immobilize PTB (Figure 2a). As clearly shown in Figure S6, The ultrasonication treated MXene in water dispersion can exfoliate MXene into a well‐dispersed, single‐layered nanoflakes. Thus, rapid and efficient bonding was facilitated between terminal hydroxyl groups (–OH) on MXene surface and the carboxyl functionalities (–COOH) from carboxylated SWCNTs, enabling bottom‐up layer‐by‐layer self‐assembly of PTB@MXSC freestanding film. Such a robust structure with redox molecules uniformly dispersed within the substrate facilitates efficient electrochemical reactions and dynamics during charge intercalation/deintercalation for energy storage [32]. To reveal the cross‐sectional morphology of PTB@MXSC composite (Figure 2b), we employed a triple ion beam system (accelerating voltage: 8 kV, cutting duration: 1.5 h) for the sample preparation with precision argon ion beam milling. PTB@MXSC exhibits a highly ordered sandwich architecture, where SWCNTs serve as interlayer pillars to expand the MXSC layered framework with ∼250 to 500 nm spacing with high structural integrity. In addition, the elemental mapping images of C, N, O, and Ti in energy‐dispersive X‐ray spectroscopy (EDS) analysis also demonstrate that PTB does not aggregate in the composite film (Figure S7). In short, SWCNTs bonded MXene formed the hierarchical reinforced concrete frameworks with PTB well distributed inside. It should be noted that precise control of the interlayer spacing (e.g., to a specific value like 100 nm) within the 3D layered conductive framework is challenging with the current ultrasonication‐assisted vacuum filtration approach, indicating a large room for future methodological optimization. Nevertheless, experimental results indicate that the interlayer spacing of the as‐prepared materials fluctuates within the range of several hundred nanometers. This scale sufficiently meets the requirements for rapid Zn^2+^ diffusion within the cathode material. Furthermore, the excellent electrochemical performance and good batch‐to‐batch reproducibility demonstrated by the PTB@MXSC composite further validate the rationality of this structural characteristic. A systematic discussion on this aspect will be presented in subsequent sections. Considering the weight ratio of PTB/MXene/SWCNTs (1:7:8), it is understood that the significantly expanded interlayer spacing disrupts the periodic stacking of MXene, leading to significantly reduced structural crystallinity as reflected by the suppressed (002) diffraction peak in XRD (Figure 1e).

TEM images reveal laterally extended MXene nanoflakes with uniform morphology and few‐layer stacking (Figure 2c). The measured interlayer spacing of 14.35 Å (Figure S8) exhibits excellent agreement with the average MXene interlayer spacing of 14.47 Å obtained from the XRD spectrum (Figure 1e), confirming its preserved crystalline lattice structure during exfoliation. However, MXene sheets is prone to aggregate due to the stacking effect between layers (Figure 2d), preventing the efficient of charge transfer between ions in electrolytes to redox active sites [38, 39]. The introduction of SWCNTs as pillars to expand the interlayer spacing of MXene via bottom‐up nanochemistry stands as a promising approach in constructing a self‐assembly hierarchical structure. PTB@MXSC freestanding film boasts homogeneous dispersion of PTB and SWCNTs fibers in 2D MXene nanosheets (Figure 2e), demonstrating excellent interfacial integration among components. The porosity of the composite was subsequently evaluated using nitrogen isothermal adsorption‐desorption measurements (Figure S9). Based on the Brunauer–Emmett–Teller (BET) model, bulky PTB exhibited a specific surface area of only 4.78 m^2^ g^−1^, whereas PTB@MXSC composite demonstrated over 39‐fold enhanced specific surface area (191.90 m^2^ g^−1^). Structural analysis indicates that the incorporation of SWCNTs effectively expands the interlayer spacing of MXene, thereby fully exposing active sites. This structural modulation is critically important for enhancing the electrochemical reaction kinetics of the hybrid material.

**FIGURE 3:**
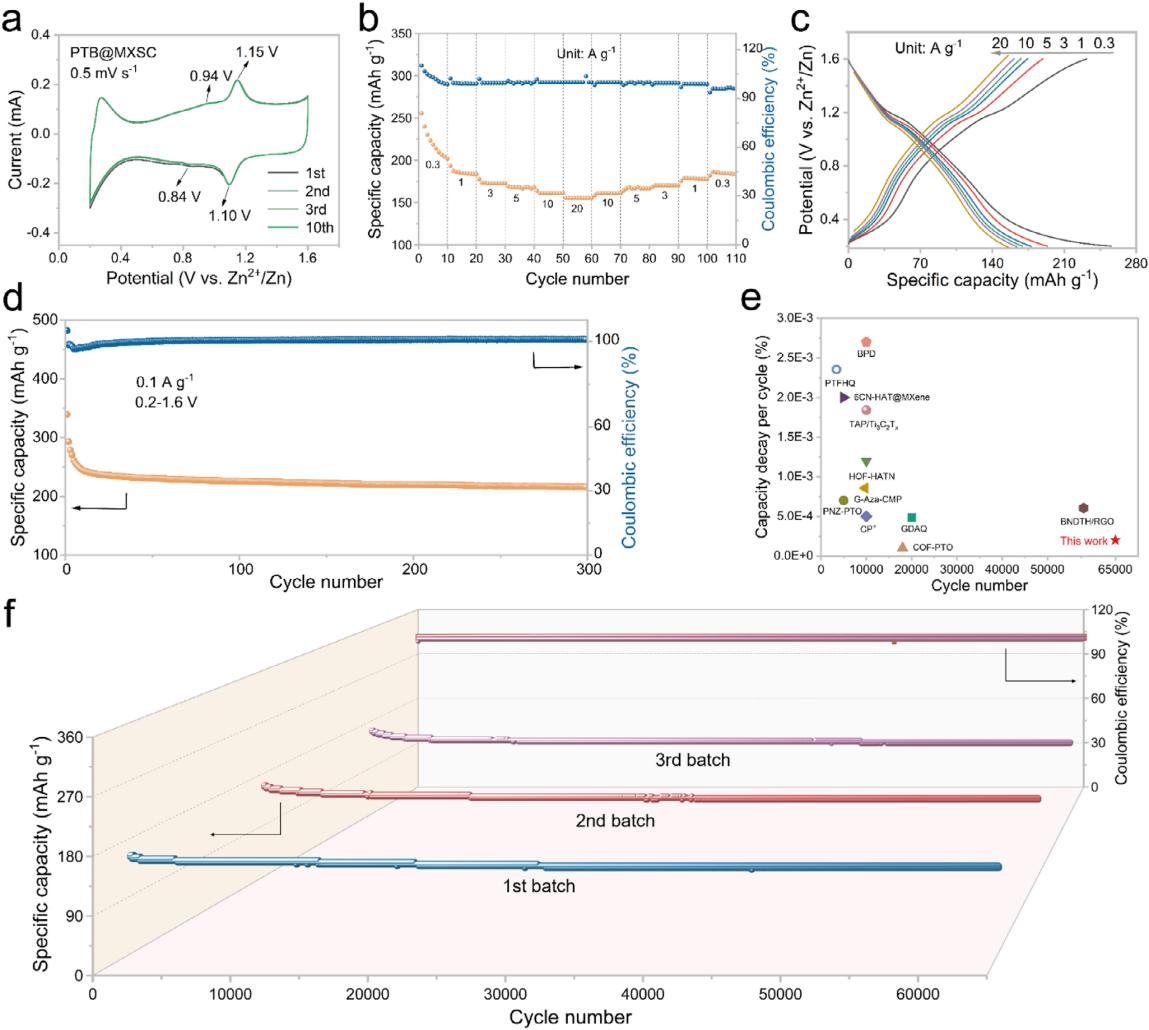
Electrochemical performance of the PTB@MXSC cathode. (a) CV curves measured at the scan rate of 0.5 mV s^−1^. (b) Rate performance in the current density range of 0.3‐20 A g^−1^. (c) GCD curves in the current density range of 0.3‐20 A g^−1^. (d) Cycling performance in the voltage range of 0.2‐1.6 V vs. Zn^2+^/Zn. (e) Comparison of the cycle number and capacity decay per cycle of different organic cathode materials in ZIBs. (f) Cycling stability of three different batches of PTB@MXSC.

The well‐defined layered architecture and homogeneous distribution of active sites in PTB@MXSC motivated our investigation into its potential application for ZIBs. Accordingly, coin‐type ZIBs were assembled using PTB@MXSC free‐standing film as cathode, zinc foil as anode, and 4 m Zn(ClO_4_)_2_ as electrolyte. To optimize the cathode composition, we systematically evaluated the cycling performance of ZIBs with PTB@MXSC cathodes by first fixing the PTB: SWCNTs composition at a varied weight ratio of MXene (Figure S10). With the better‐performing PTB:MXene: SWCNTs (1:7:6) at hand, we further optimized the cathode composition by varying the weight ratio of SWCNTs. Among all nine tested cathodes, PTB@MXSC with PTB:MXene: SWCNTs weight ratio as 1:7:8 demonstrated the best cycling stability, as reflected by 81.18% exceptional capacity retention after 5000 cycles at 3 A g^−1^. This optimal composition was consequently secured for the cathode for systematic investigations. To validate the rational design of PTB molecules, BQ@MXSC and PA@MXSC free‐standing film cathodes with the same composition were prepared as a reference to PTB@MXSC (Figure S11), where BQ or PA served as the redox molecules and MXSC as the conductive framework. The electrochemical properties of three free‐standing cathode films were initially characterized by cyclic voltammetry (CV) at a scan rate of 0.5 mV s^−1^ within a potential window of 0.2–1.6 V (versus Zn^2+^/Zn). The measurements were continuously conducted over multiple cycles to evaluate their redox behaviour and cycling stability (Figure 3a; Figure S12). The nearly superimposable CV curves of PTB@MXSC over cycling indicate high reversible redox processes, where two pairs of redox peaks are observed at 1.15/1.10 and 0.94/0.84 V, corresponding to the dual active sites within the PTB molecular framework. In contrast, PA@MXSC shows only one pair of redox peaks at 1.03/0.95 V assigned to C═N functionality, while BQ@MXSC exhibits one well‐defined redox pair at 1.16/1.08 V attributed to the redox reaction of C═O. Notably, during the discharge process, the reduction potential of the organic cathode exhibits a negative correlation with its LUMO energy level, i.e., lower LUMO, higher reduction potential, which reflects the molecule's enhanced electron affinity and thermodynamic tendency toward reduction [40, 41, 42]. As illustrated in Figure 1b, PTB exhibits decreased LUMO energy levels than PA and BQ, thus correlating well with the increased positive reduction potentials. BQ@MXSC showed dramatically enhanced integrated areas under CV traces than PA@MXSC and PTB@MXSC, indicating its improved specific capacity. This result aligns well with the theoretical capacity calculated for BQ (495.9 mAh g^−1^), PA (297.5 mAh g^−1^) and PTB (409.9 mAh g^−1^), respectively.

The rate stability of PTB@MXSC was evaluated in a wide current density range from 0.3 to 20 A g^−1^. As illustrated in Figure 3b,c, PTB@MXSC cathode delivered stable and reversible specific capacity of 255, 194, 178, 169, 164, and 155 mAh g^−1^ at the corresponding current density of 0.3, 1, 3, 5, 10, and 20 A g^−1^ each for 10 cycles, respectively. In addition, the slow decay in specific capacity for PTB@MXSC cathode with increasing current density underscores its exceptional charge‐transfer kinetics, robust structural integrity, and superior interfacial stability, which collectively contribute to its extraordinary high‐rate cycling performance.

Comparison study for PA@MXSC and BQ@MXSC could only be evaluated within a narrower current density region of 0.3–10 A g^−1^ (Figures S13 and S14). Notably, PA@MXSC exhibits better cycling stability than BQ@MXSC, which may be ascribed to the differences of PA and BQ solubility in aqueous electrolytes (as will be discussed in detail later). Interestingly, BQ@MXSC displays a well‐defined discharge plateau at ∼1.16 V in galvanostatic charge/discharge (GCD) measurement, while both PTB@MXSC and PA@MXSC show less pronounced potential profiles during the discharge process. These observations align well with their redox behaviors observed in the CV test (Figure 3a; Figure S12), further corroborating the electrochemical characteristics of n‐type quinones and A‐D‐A molecule systems. The cycling performance of PTB@MXSC was evaluated within a potential window of 0.2–1.6 V or 0.2–1.8 V at a current density of 0.1 A g^−1^ (Figure 3d). When cycled between 0.2–1.8 V, PTB@MXSC delivered an ultrahigh initial specific capacity of 407.6 mAh g^−1^ at 0.1 A g^−1^, but the Coulombic efficiency gradually increased from 70% to 100% over the first 50 cycles before leveling off. Its long‐term cycling stability in the potential window of 0.2–1.8 V under high current density (ca. 10 A g^−1^) remained unsatisfactory, e.g., only a 64.8% capacity retention was achieved after 800 cycles (Figure S15). This rapid capacity decay in the high potential window may be attributed to irreversible electrode structural degradation caused by excessive cation intercalation during the discharge process [43]. Excitingly, under the optimized potential window of 0.2–1.6 V, PTB@MXSC cathode delivers an impressive reversible capacity of 339.5 mAh g^−1^ at 0.1 A g^−1^ while maintaining near‐unity Coulombic efficiency over 300 charge/discharge cycles, demonstrating exceptional electrochemical cycling stability.

Remarkably, the well‐defined hierarchical nanostructure and optimal potential window endow PTB@MXSC with superior reproducibility. For instance, PTB@MXSC//Zn coin cells made from three independently prepared batches of cathodes delivered outstanding long‐term cyclability with an average capacity retention of 87.5% after 65 000 cycles at a high current density of 10 A g^−1^ (Figure 3f), corresponding to an ultralow average capacity decay rate of merely 1.9 × 10^−4^ percent per cycle. In particular, PTB@MXSC//Zn delivers the highest energy density of 244.63 Wh kg^−1^ at a power density of 72.16 W kg^−1^, surpassing the performance of typical organic cathodes for ZIBs (ca. HATN‐3CN, HAQ‐COF, TAP/Ti_3_C_2_T_x_ and AOPs) reported in the literature (Figure S16a). Moreover, PTB@MXSC contributes the highest specific capacity of 339.5 mAh g^−1^ at 0.1 A g^−1^ and over 150 mAh g^−1^ at 20 A g^−1^, outperforming the representative ZIBs cathodes (ca. C4Q, HOF‐HATN, PNZ‐PTO,

AOPs, CP^+^, COF‐PTO) in the literature (Figure S16b). Notably, PTB@MXSC exhibits extraordinary cycling stability, ranking the best‐performing organic cathodes reported for ZIBs to date (Figure 3e), including hexaazatriphenylene hexacarbonitrile@MXene (6CN‐HAT@MXene) [44], imine‐based tris(aza)pentacene (TAP/Ti_3_C_2_T_x_) [32], COF containing pyrene‐4,5,9,10‐tetraone groups (COF‐PTO) [35], and others [18, 36, 45, 46, 47, 48, 49, 50].

To reveal the impact of molecular structure on the electrochemical stability of organic redox cathode, a comparison study was conducted to evaluate the long‐term cycling performance of PA@MXSC and BQ@MXSC under the same test conditions as for PTB@MXSC at a current density of 10 A g^−1^ (Figure S17). PA@MXSC also exhibits good cycling stability, with 91.52% of its initial capacity retained after 10 000 cycles, while BQ@MXSC displays undergoes this behaviour, which can be explained by the severe dissolution of BQ in aqueous 4 m Zn(ClO_4_)_2_ electrolyte, as indicated by the obvious colour change within 30 days of storage (Figure S18a). In addition, post‐cycling disassembly revealed that the separator in the PTB@MXSC//Zn cell remained clean, with no noticeable discoloration arising from dissolved active species (Figure S18b). In contrast, the separator from the BQ@MXSC//Zn cell exhibited pronounced coloration, providing direct evidence of the substantially lower solubility of PTB. The comparative analysis of the morphology (SEM), electrochemical behavior (CV), and molecular structure (^1^H NMR) of the PTB molecule before and after extended cycling collectively demonstrates that its chemical integrity remains fully preserved even under harsh electrochemical cycling conditions (Figure S19).

To elucidate the impact of substrate on the electrochemical performance of PTB@MXSC, a comparison study was conducted with a conventional cathode (denoted as PTB@CP) by blending 60 wt.% PTB with 30 wt.% carbon black and 10 wt.% polytetrafluoroethylene (PTFE). As illustrated in Figure S20a, the PTB@CP cathode fabricated using stainless‐steel mesh as the current collector was assembled into a PTB@CP//Zn coin cell for electrochemical evaluation. PTB@CP exhibits a similar CV profile to that of BQ@MXSC (Figure S20b), but with significantly lower active material utilization. Notably, the CV curve of PTB@CP displays an anodic peak shift toward higher potentials and a cathodic peak shift toward lower potentials, indicative of a higher energy barrier for electrochemical reactions and weaker electron affinity [18, 51]. These factors collectively contribute to the poor cycling stability and reversibility of PTB@CP cathode at even low current density like 0.1 A g^−1^ (Figure S20c). The inhomogeneous distribution of fluorine atoms observed in EDS mapping of PTB@CP clearly indicates the aggregation of PTFE binder within the electrode (Figure S21). This phase segregation leads to two detrimental effects: (i) compromised utilization of redox molecules due to blocked ionic/electronic pathways, and (ii) significantly increased electrode‐electrolyte interface impedance resulting from poor wettability and discontinuous charge transport networks (Figures S22 and S23). Impedance analysis reveals that the performance disparity between PTB@MXSC and PTB@CP originates from fundamental differences in their charge‐storage mechanisms (Table S1). Although the two electrodes exhibit comparable charge‐transfer resistance (R_ct_ values of 137 Ω and 211.9 Ω, respectively), suggesting similar intrinsic electrochemical reaction kinetics, the key distinction lies in their mass‐transport behavior. The lower finite‐length diffusion resistance (W_o_‐R = 285.3 Ω) of PTB@CP typically corresponds to a thinner or more densely packed active material layer with limited accessible reaction interfaces. Therefore, the charge storage is mainly concentrated in the near‐surface region. This is consistent with the lower electrochemical energy storage capacity mentioned earlier (Figure S20c). In contrast, PTB@MXSC demonstrates a significantly higher finite‐length diffusion resistance (W_o_‐R = 392.4 Ω), reflecting the extended transport pathways for ions within its well‐developed 3D layered network. However, coupled with its markedly lower R_ct_, this indicates that the MXSC scaffold creates a substantially enlarged electrochemically active interface. Consequently, the dominant storage mechanism shifts from sluggish bulk diffusion control to a hybrid process driven by rapid surface‐mediated processes and highly efficient charge transfer. Thus, despite possessing comparable intrinsic reaction kinetics, PTB@MXSC achieves superior active material utilization and enhanced overall mass‐transport efficiency. To elucidate the reasons of the superior electrochemical performance of PTB@MXSC, we systematically investigated its redox reaction kinetics through CV measurements at varied scan rates from 0.1 to 3 mV s^−1^. As demonstrated in Figure S24a, the CV curves maintain two well‐defined redox couples with only marginal polarization effects when the scan rate increases from 0.1 to 2 mV s^−1^, indicating highly reversible charge storage processes and favorable reaction kinetics in the PTB@MXSC electrode [52].

The redox reaction kinetics of the PTB@MXSC cathode was quantitatively analyzed by examining the power‐law relationship between the peak current (*i*) and scan rate (*v*) as *i = av^b^*, where *a* and *b* are adjustable parameters [53]. Their value serves as the diagnostic parameter to determine the dominant charge storage mechanism, i.e., *a* value of 0.5 indicates a diffusion‐controlled process governed by bulk ion transport limitations, while *b* = 1.0 corresponds to ideal capacitive behaviour dominated by surface charge adsorption. The *b* value can be obtained by fitting the linear relationship between log(*i*) and log(*v*). Electrochemical kinetic analysis of PTB@MXSC reveals near‐ideal capacitive behaviour, as evidenced by the calculated *b*‐values of 0.973, 0.956, 0.955, and 0.970 for the two redox couples in Figure S24b. All *b* values are close to 1, indicating that the capacitive behaviour dominates the reaction process and therefore has a faster ion transfer rate. The capacitive contribution increases with the increase of scanning rate, with 88% reached at an elevated scan rate of 3 mV s^−1^ (Figure S24c,d). The galvanostatic intermittent titration technique (GITT) analysis reveals that the PTB@MXSC electrode achieves a superior ion diffusion coefficient in the range of 10^−8^‐10^−7^ cm s^−1^ (Figure S25), surpassing most reported organic cathode materials for ZIBs to date (Table S2). The reaction kinetics of PTB@CP reveal a larger integrated area under the reduction peak in CV curves than under the oxidation peak, indicating a higher discharge capacity than charge capacity for the PTB@CP//Zn cell (Figure S26). This observation aligns with the consistently observed Coulombic efficiency (CE) exceeding 100% in the PTB@CP//Zn coin cell. In addition, PTB@CP was determined to have a significantly higher capacitive contribution than PTB@MXSC, from quantitative kinetics analysis, which can be explained with its too small specific surface area and poor electrode‐electrolyte interface contact to expose enough active sites for redox reaction. We attribute these limitations primarily to the aggregation of PTFE binder within slurry‐coated electrode architecture, which inevitably impedes bulk‐phase electrochemistry by creating physical barriers to ion transport and active material accessibility.

We also evaluated the capacity contribution from the MXSC substrate (MXene: SWCNTs = 7:8, weight ratio) as cathode (Figure S27). MXSC can only deliver a specific capacity of 37.5 mAh g^−1^. This remarkably low capacity output unequivocally indicates that the overwhelming majority of the capacity achieved by PTB@MXSC originates from PTB active material, rather than the MXene‐SWCNTs conductive framework. The weak capacity contribution from MXSC further highlights that PTB is the primary active component responsible for enabling substantial charge storage within the hierarchical nanostructure. Further investigation into PTB@MXSC freestanding film cathodes revealed a strong dependence of capacity upon PTB content in the composites. When doubling PTB loading, PTB@MXSC (PTB:MXene: SWCNTs = 2:7:8, weight ratio) demonstrates clear PTB aggregation within the MXSC matrix from SEM observation (Figure S28), which is not conducive to take full advantage of active functional sites. This reflects on 5000‐cycle cycling performance with the initial capacity of only 94.2 mAh g^−1^ decay to 64.2 mAh g^−1^ at 3 A g^−1^ (Figure S29). These results highlight the critical balance between active material loading and structural homogeneity in hybrid cathode design, where excessive PTB incorporation compromised the electrochemical performance of cathodes. To further substantiate the superior performance of the PTB@MXSC freestanding film cathode, we evaluated its compatibility with aqueous zinc salt‐based electrolytes using three representative ones, including 2 m ZnSO_4_, 2 m Zn(OTf)_2,_ and 2 m Zn(CH_3_COO)_2_ (Figure S30). Remarkably, PTB@MXSC//Zn coin cells exhibit exceptional cycling stability in all electrolytes upon cycling to 2500 cycles at 3 A g^−1^ (ca. 100% Columbic efficiency), underscoring their outstanding compatibility with diverse electrolytes. This observation also suggests the charge storage is predominantly governed by the stable redox chemistry of PTB rather than specific ion‐electrode interactions. These findings significantly expand the practical applicability of this cathode for energy storage in ZIBs.

To further approach practical application conditions, we increased the areal loading of the active material while maintaining the same component ratio. When the areal loading was raised from 1.0 to 2.3 mg cm^−2^, with the PTB:MXene: SWCNT mass ratio fixed at 1:7:8, a beneficial trade‐off was observed (Figure S31). At a current density of 3 A g^−1^, the electrode delivered an initial capacity of 173 mAh g^−1^ and retained 92% of its capacity after 1000 cycles. Although a slight decrease in specific capacity occurred, the long‐term cycling stability was substantially enhanced. This improvement is likely associated with the increased electrode thickness, which introduces mild ion‐diffusion limitations and reduces the full utilization of active sites, thereby resulting in a marginal capacity decrease. Meanwhile, the higher mass loading facilitates the formation of a more robust 3D conductive and mechanical network, strengthening the overall structural integrity of the electrode. Consequently, this minor sacrifice in mass‐specific capacity yields notable improvements in structural stability and cycling lifespan, which are of greater importance for practical applications, thereby demonstrating the excellent scalability of the PTB@MXSC composite electrode design.

**FIGURE 4:**
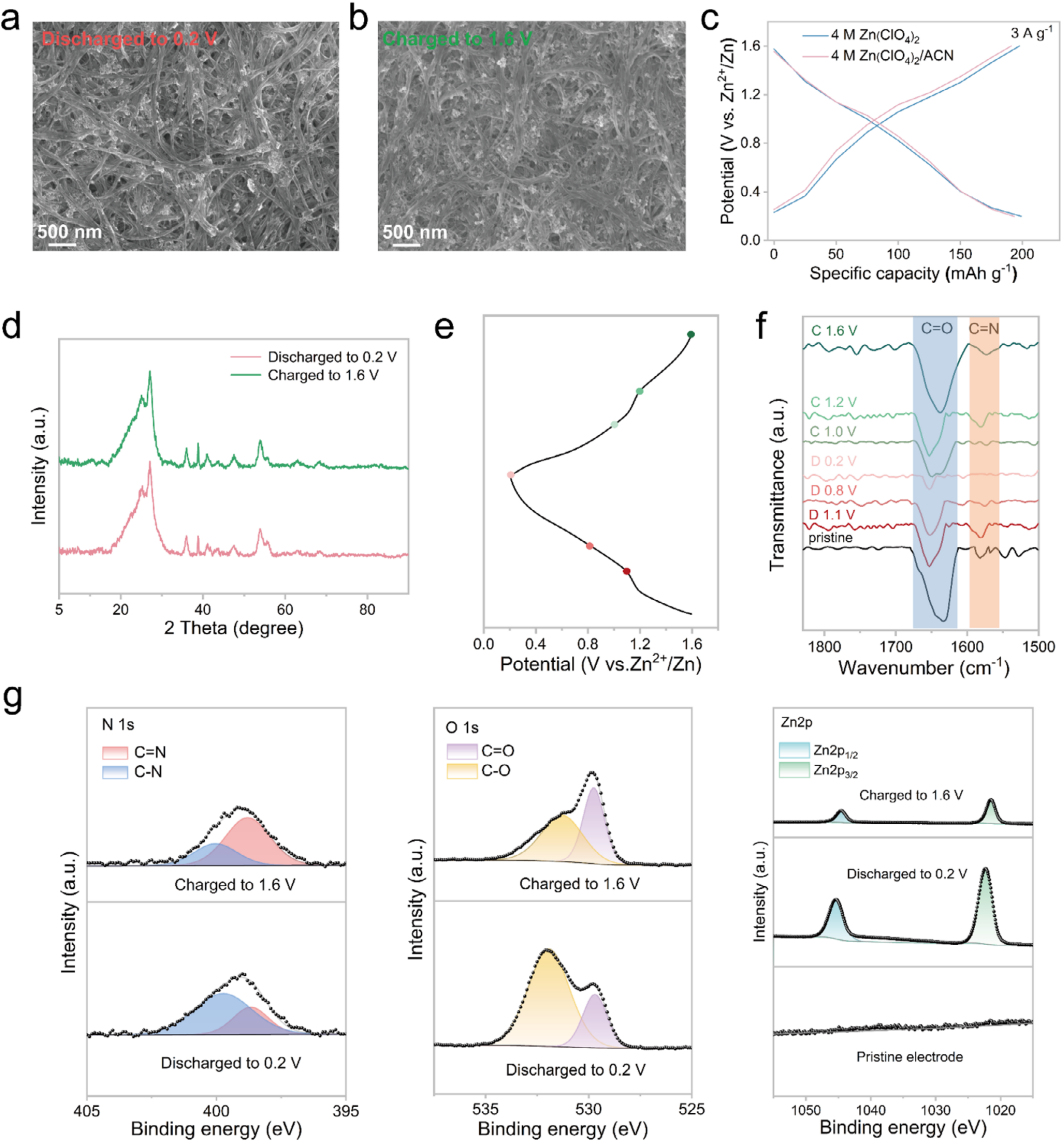
SEM images of PTB@MXSC cathode at (a) fully discharged (0.2 V) and (b) fully charged (1.6 V) states. (c) GCD curves of PTB@MXSC in 4 m Zn(ClO_4_)_2_ and 4 m Zn(ClO_4_)_2_/ACN electrolytes at 3A g^−1^. (d) Ex situ XRD patterns of PTB@MXSC cathode at fully discharged (0.2 V) and fully charged (1.6 V) states. (e) GCD curves, (f) The ex situ FTIR spectra, and (g) XPS spectra of N 1s, O 1s, and Zn 2p at the marked points of PTB@MXSC cathode during charge/discharge processes.

To gain deeper insights into the electrochemical energy storage mechanism of PTB@MXSC, a series of ex situ structural characterizations were systematically conducted. As revealed from the SEM images (Figure 4a,b), PTB molecules remain homogeneous distribution as non‐aggregated granular domain in hierarchical nanostructure after PTB@MXSC cathode discharged to 0.2 V and subsequently charged to 1.6 V. Further EDS mapping analysis provides compelling evidence for the reversible Zn^2+^ intercalation/deintercalation process during discharge/charge cycles, i.e., successful Zn^2+^ insertion into PTB@MXSC upon discharging and subsequent extraction during charging stages (Figure S32). Quantitative elemental analysis of the PTB@MXSC cathodes reveals that the Zn/N molar ratio increases from approximately 0.48 in the fully charged state to 1.57 in the fully discharged state(Table S3 and Figure S33). This value is in excellent agreement with the theoretical ratio of 1.5, which corresponds to the coordination of six Zn^2^⁺ ions per two PTB molecules, thereby unequivocally identifying Zn^2^⁺ as the primary charge carrier in the PTB redox reaction. PTB@MXSC cathode exhibits negligible structural variations in XRD patterns from fully charged to discharged states, suggesting the absence of significant phase transitions during electrochemical cycling (Figure 4d). This observation strongly indicates that H⁺ are unlikely to participate in the energy storage mechanism, further supporting the dominant role of Zn^2+^ insertion/extraction in the redox process. This phenomenon can be attributed to the significantly higher concentration of Zn^2+^ compared to H⁺ in 4 m Zn(ClO_4_)_2_ electrolyte [44]. To validate this hypothesis, PTB@MXSC were performed in an aprotic electrolyte system consisting of 4 m Zn(ClO_4_)_2_/acetonitrile (4 m Zn(ClO_4_)_2_/ACN) for electrochemistry. As evidenced by the GCD profiles in Figure 4c, the PTB@MXSC electrode delivers comparable specific capacity (ca. 193.5 mAh g^−1^) in 4 m Zn(ClO_4_)_2_/ACN electrolyte to that in aqueous 4 m Zn(ClO_4_)_2_, while maintaining similar CV curve characteristics (Figure S34). These consistent electrochemical behaviors across fundamentally different electrolyte environments strongly suggest that Zn^2+^ insertion/extraction represents the dominant charge storage mechanism, with negligible contribution from proton‐mediated processes. Interestingly, when strong acidic aqueous electrolytes were employed (ca. pH 1 H_2_SO_4_ solution), PTB@MXSC exhibited a comparable capacity to that observed in 4 m Zn(ClO_4_)_2_ (pH 0.62), suggesting its potential applicability in proton‐based battery systems (Figure S35). This observation implies that both C═N and C═O redox‐active sites in the PTB framework can effectively accommodate H⁺ insertion in the absence of Zn^2+^ (Figure S36). The dual‐ion storage capability highlights the structural versatility of PTB@MXSC as a promising cathode for both zinc‐ion and proton‐based energy storage devices.

To gain mechanistic insights into Zn^2+^ storage behaviour, we performed systematic ex situ FTIR characterization of PTB@MXSC electrodes at different charge/discharge states (Figure 4e,f). The spectral evolution reveals reversible intensity changes of characteristic vibrational modes at 1650 cm^−1^ (C═O) and 1580 cm^−1^ (C═N), showing progressive weakening during discharge to 0.2 V and complete recovery upon charging to 1.6 V. The FTIR spectrum of the pristine PTB@MXSC cathode closely matches that after charging to 1.6 V, while clearly differing from the spectrum after discharging to 0.2 V. This phenomenon can be attributed to the reversible insertion/deinsertion of Zn^2+^ in the C═O and C═N active sites. To further elucidate the Zn^2+^ storage mechanism, ex situ XPS analysis was employed to track the evolution of key functional groups during electrochemical cycling (Figure 4g). High‐resolution N 1s and O 1s spectra reveal distinct changes in the chemical environment of PTB@MXSC: during discharge to 0.2 V, the characteristic peaks of C═O (529.8 eV in O 1s) and C═N (398.8 eV in N 1s) show significant attenuation while new components emerge at higher binding energies (O 1s: 531.9 eV; N 1s: 399.7 eV), corresponding to the formation of C─O─Zn and C─N─Zn coordination bonds. This reversible change in bond strength during charge and discharge is also found in the XPS spectrum of the Zn 2p peak, indicating that Zn^2+^ can be reversibly inserted/deinserted in C═O and C═N during (dis)charge process. This is consistent with the previous conclusion. These XPS observations provide direct spectroscopic evidence for the proposed redox mechanism involving reversible Zn^2+^ binding at carbonyl and imine functional groups.

The combined XRD, FTIR, and XPS analyses have confirmed the reversible Zn^2^⁺ insertion/extraction behavior in the PTB@MXSC composite from a chemical perspective. To further explore the structural root of its cyclic stability, we conducted a cross‐sectional morphology analysis on the PTB@MXSC cathode after 65 000 cycles at 10 A g^−1^ (Figure S37). SEM observations reveal that the microstructure of the material remains well‐preserved. The MXene sheets exhibit no collapse or excessive restacking, maintaining an intact layered framework, while the SWCNTs remain firmly bonded to the MXene without noticeable detachment. These results directly demonstrate the excellent mechanical and structural stability of the 3D conductive scaffold, which effectively accommodates cyclic stress and mitigates electrode pulverization, thereby providing a durable and stable host for the reversible Zn^2+^ intercalation chemistry. This robust structural integrity serves as a critical foundation for achieving the outstanding long‐term cycling performance of the electrode.

**FIGURE 5:**
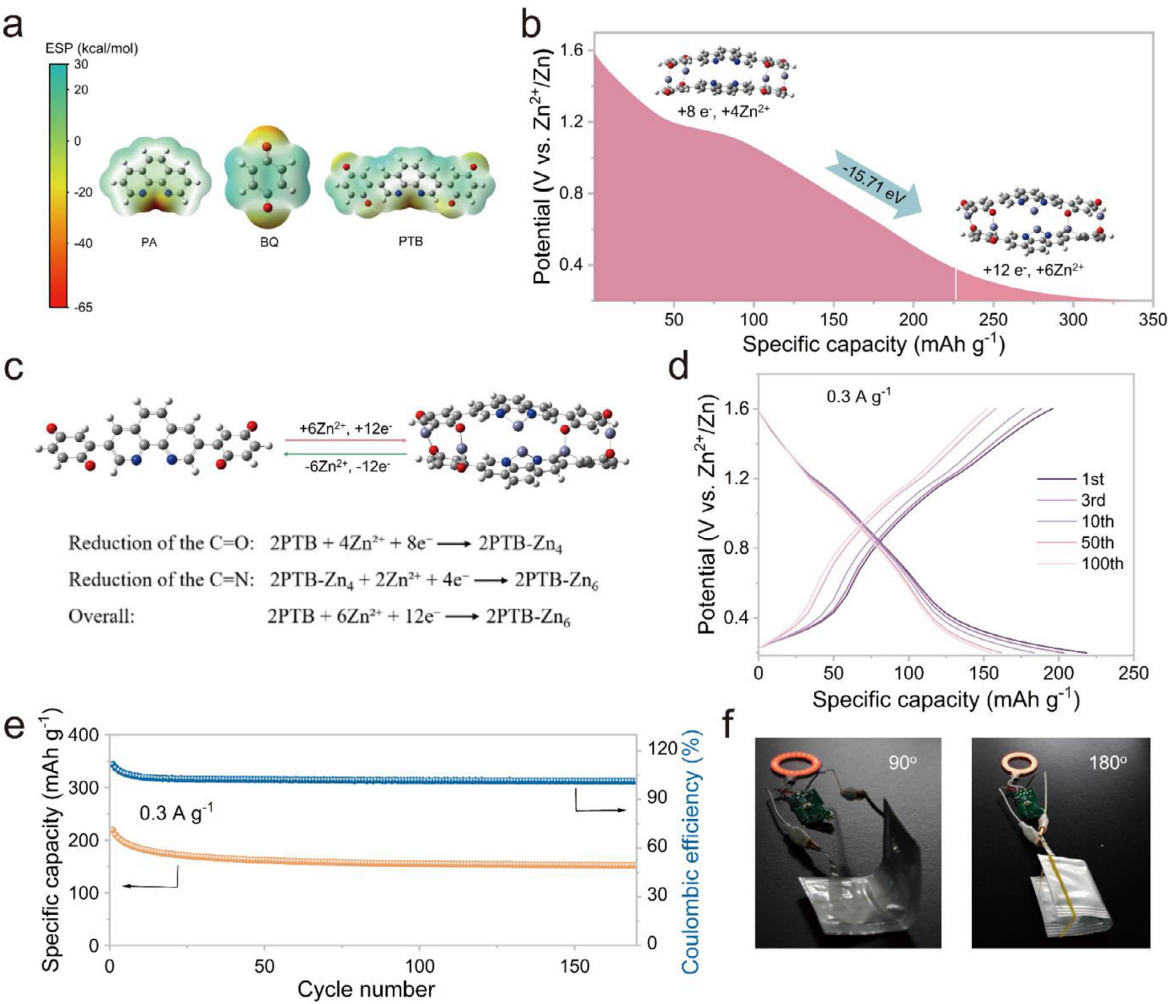
(a) The ESP mapping of the molecular van der Waals surface for PTB and its two fragment molecules. (b) Calculated possible structural evolution and Zn^2+^ insertion pathway at discharging process, shown with the calculated ΔG values. (c) Optimized configurations of PTB before and after Zn^2+^ ion uptake. Pouch cell performance: (d) Charge/discharge curves under different cycles. (e) Cycling performance at 0.3 A g^−1^. (f) Working state of ZIBs under different bending angles.

To clarify Zn^2+^ storage mechanism in the PTB@MXSC cathode, molecular electrostatic potential (MESP) analysis was used to visualize the active sites. As illustrated in Figure 5a, the carbonyl oxygen and imine nitrogen atoms exhibit lower MESP values (red regions) with decreasing electronegativity, revealing pronounced negative charge localization. This observation suggests preferential Zn^2+^ electrophilic interactions at these electron‐rich sites during discharge, unequivocally identifying both C═O and C═N groups as dual redox‐active centers within the PTB framework. The Mulliken charges can be used to describe the electron density enrichment on the target atom [54], so we calculated the Mulliken charge values of nitrogen and oxygen atoms in PTB. As depicted in Figure S38, the Mulliken charge values of O atoms on benzoquinone in different chemical environments are −0.231 and −0.253, respectively, while the Mulliken charge value of the N atom on the imine group is −0.101. The significant difference in Mulliken charges between the heteroatoms suggests that the C═O groups in PTB possess higher redox activity than the C═N moieties, which means that during the discharge process, Zn^2+^ ions preferentially coordinate with the C═O sites first. This computational finding aligns well with the electrochemical behaviour observed in CV profiles of PTB@MXSC, where the redox peaks corresponding to the quinoid oxygen units appear at a higher potential than those of the imine nitrogen sites. The aforementioned ex situ infrared spectroscopy (Figure 4f) analysis also confirms the plausibility of the “stepwise storage mechanism”: within the higher voltage range (approximately 1.2–1.0 V versus Zn^2+^/Zn), the C═O sites, which possess stronger electronegativity, preferentially bind with Zn^2+^, leading to prominent and early changes in their infrared signals. In contrast, the C═N sites remain largely inactive at this stage, exhibiting only minor spectral responses. This spectroscopic evidence further supports the sequential Zn^2+^ storage mechanism in PTB, in which insertion proceeds stepwise with C═O sites engaged prior to C═N sites.

Subsequently, we systematically investigated the thermodynamic feasibility of cation intercalation by evaluating the possible insertion pathways at different active sites, accompanied by quantitative analysis of the corresponding Gibbs free energy changes (Figure S39). The calculation results reveal that the Zn^2+^ coordination complex formed between four Zn^2+^ ions and eight C═O groups from separate PTB molecules exhibits greater stability (ΔG = −31.89 eV) than complexes involving C═N coordination (ΔG = ‐6.35 eV). This bidentate binding mode aligns with previously reported metal‐coordination behaviour in similar quinoid molecules [55]. The same situation also appears in the difference in Gibbs free energy changes after C═O and C═N in a PTB molecule bind to H^+^. The higher stability of C═O after binding to cations indicates that C═O has higher redox activity than C═N, as evidenced by both computational charge distribution analysis and electrochemical characterization. Although the stability of each PTB molecule binding to 6 H^+^ is higher than that of other Zn^2+^‐containing configurations, considering that the concentration of Zn^2+^ in the actual electrolyte is much higher than that of H^+^, the possibility of this configuration existing is relatively low. Based on our experimental and computational analyses, we propose a two‐step, six‐electron storage mechanism for PTB@MXSC involving sequential Zn^2+^ coordination: first through the cooperative binding of four Zn^2+^ ions with eight C═O from adjacent PTB molecules, followed by the subsequent association of two additional Zn^2+^ ions with four C═N, ultimately forming the stable discharge product 2PTB‐Zn_6_ (Figure 5b,c). In the PTB@MXSC cathode, only Zn^2^⁺ participates in the charge storage process, and thus, the contribution of each Zn^2^⁺ binding to an active site to the overall capacity is uniform. Specifically, the C═O contributes two‐thirds of the total capacity, while the C═N accounts for the remaining one‐third.

Leveraging the superior electrochemical properties of the coin cell configuration, we fabricated a flexible pouch cell to demonstrate the practical utility of PTB@MXSC. As depicted in Figure 5d, the PTB@MXSC//Zn pouch cell delivers an initial capacity of 218.6 mAh g^−1^ at 0.3 A g^−1^, coupled with remarkable cycling stability (Figure 5e). In addition, we have supplemented the pouch‐cell evaluation with rate‐performance tests and provided GCD curves obtained at different current densities (Figure S40). The results show that the pouch cell maintains well‐defined voltage plateaus across all tested current densities and achieves nearly 100% Coulombic efficiency, confirming its excellent rate capability and electrochemical reversibility under practical device configurations. Notably, the fully charged PTB@MXSC//Zn pouch cell can replace a commercial No.7 battery to power a small electronic device even when subjected to bending at various angles (0‐180°) (Figure 5f; Video S1). These results demonstrate its promising potential for application in flexible electronic devices.

## 3 Conclusion

In summary, we have successfully developed an electron acceptor‐donor‐acceptor redox molecule to immobilize into a conductive platform imitating reinforced concrete framework as freestanding cathodes for ultrastable high‐performance aqueous zinc‐ion batteries. This rational design and solution assembly approach endows the synergistic interplay between active‐sites rich redox molecule and the 3D hierarchical nanostructure of layered MXene and SWCNTs network to secure both structural integrity and efficient electrochemistry. As a result, the optimal cathode exhibited the highest specific capacity of 339.5 mAh g^−1^ at 0.1 A g^−1^, together with a superior capacity retention of 87.5% after 65 000 cycles at 10 A g^−1^ current density. Electrochemical analysis reveals that the optimal cathode has a high Zn^2+^ diffusion coefficient ranging from 10^−8^ to 10^−7^ cm^2^ s^−1^ to facilitate ion transport kinetics. Systematical spectroscopic characterization and theoretical calculations elucidate a unique two‐step six‐electron‐involving redox mechanism for Zn^2+^ storage within the hierarchical nanostructure. The remarkable structural robustness of the hybrid cathode during charge/discharge cycles highlights its potential as unltrastabke cathode material for Zn‐ion storage systems. Therefore, the strategy of immobilizing acceptor‐donor‐acceptor organic molecules with maximal redox active sites into conductive hierarchical architecture in this work opens a new avenue to pursue high‐performance ultrastable cathodes for aqueous zinc‐ion batteries.

## Conflicts of Interest

The authors declare no conflicts of interest.

## Supporting information




**Supporting File**: advs73609‐sup‐0001‐SuppMat.docx.

**Supporting File**: advs73609‐sup‐0002‐Video S1.mp4.

## Data Availability

The data that support the findings of this study are available in the Supporting Information of this article.
