# Supplementary material for: Redox Oligomer Assembling Hierarchical Reinforced Framework Cathodes for Ultra‐Stable High‐Performance Zinc‐Ion Batteries
Source: Adv Sci (Weinh). 2026 Jan 4;13(14):e22537. doi: 10.1002/advs.202522537 (PMC12970184; doi:10.1002/advs.202522537)
Supplement: Supplementary file 1 — Supporting File: advs73609‐sup‐0001‐SuppMat.docx. [file ADVS-13-e22537-s002.docx]

Supporting Information

Redox Oligomer Assembling Hierarchical Reinforced Framework Cathodes for Ultra-Stable High-Performance Zinc-Ion Batteries

*Shuang Liu, Yiliang Lai, Yinghang Gao, Weihua Tang**

S. Liu, Y. Lai, W. Tang

College of Materials, College of Chemistry and Chemical Engineering, Innovation Laboratory of Energy Materials of Fujian Province (IKKEM), Xiamen University, Xiamen 361005, Fujian, China

Y. Gao

College of Energy, Xiamen University, Xiamen 361005, Fujian, China

E-mail: whtang@xmu.edu.cn

**Experimental Section**

**Materials**

Commercially availabe 3,8-dibromo-1,10-phenanthroline (≥96%), (2,5-dimethoxyphenyl)boronic acid (≥98%), acetonitrile (ACN, ≥99.9%), phenanthroline (PA, ≥99%), benzoquinone (BQ, ≥99.5%), zinc perchlorate hexahydrate (Zn(ClO_4_)_2_⋅6H_2_O, ≥99.9%), Ceric ammonium nitrate (Ce(NH_4_)_2_(NO_3_)_6_, ≥99.9%) and polytetrafluoroethylene (PTFE, BR) were purchased from Shanghai Aladdin Biochemical Technology Co., Ltd. Zinc sulfate (ZnSO_4_, ≥99%), zinc acetate (Zn(CH_3_COO)_2_, ≥99%) and zinc trifluoromethanesulfonate (Zn(CF_3_SO_3_)_2_, ≥99%) were purchased from Adamas-beta®. Carboxylated single-walled carbon nanotubes (SWCNTs) was purchased from XFNANO. Zinc foil was purchased from Canrd Technology Co. Ltd.

**Preparation of 2,2'-(1,10-phenanthroline-3,8-diyl)bis(cyclohexa-2,5-diene-1,4-dione) (PTB)**.

2,2'-(1,10-Phenanthroline-3,8-diyl)bis(cyclohexa-2,5-diene-1,4-dione) (PTB) was obtained by Suzuki coupling reaction between 3,8-dibromo-1,10-phenanthroline-5,6-dione and (2,5-dimethoxyphenyl)boronic acid and a followed demethylation via 3,8-bis(2,5-dimethoxyphenyl)-3,4-dihydro-1,10-phenanthroline (BDP) as the key intermidiate compound.^1^ The procedure was elaborated as following.

Firstly, towards a dry 100 mL three-necked round-bottom flask was added in sequence with 3,8-dibromo-1,10-phenanthroline (2.0 mmol, 676.0 mg), 2,5-dimethoxyphenylboronic acid (5.0 mmol, 909.9 mg) and K_2_CO_3_ (5.0 mmol, 691.0 mg). Subsequently, 30 mL of toluene and tetrakis(triphenylphosphine)palladium (0.1 mmol, 115.5 mg) were added. And the mixture was stirred at 110 ^o^C and nitrogen atmosphere for 24 h. After the reaction was completed, the solution was vacuum-filtered to obtain a solid powder, which was then washed with water three times to obtain the intermediate product BDP.

The dried BDP was dissolved in 30 mL of acetonitrile. The mixture solution was then placed in an ice bath and cooled to 0 ^o^C. To the solution was then slowly added with 10 mL of 0.4 M cerium ammonium nitrate aqueous solution. After warmed back to room temperature, the reaction was continued for 8 h. before terminated by deionized water. The product was filtered and washed three times with water, and then dried to obtain the final product PTB as a brown solid (613.67 mg, yield 78.2%).^1^H NMR (500 MHz, DMSO-*d*_6_) δ: 8.46 (d, J = 8.4 Hz, 2H), 8.21 (s, 2H), 7.95 (d, J = 8.2 Hz, 2H), 7.13 (s, 2H), 7.04 (d, J = 10.1 Hz, 2H), 6.97 (d, J = 10.5 Hz, 2H). ^13^C NMR (125 MHz, CF_3_COOD) δ: 189.44, 186.62, 147.59, 143.62, 141.68, 137.85, 137.65, 136.76, 136.03, 131.68, 131.06, 129.25. MALDI-TOF (m/z) calculated for C_24_H_12_N_2_O_4_ [M+H]^+^: 393.0870, found, 393.0875.

**
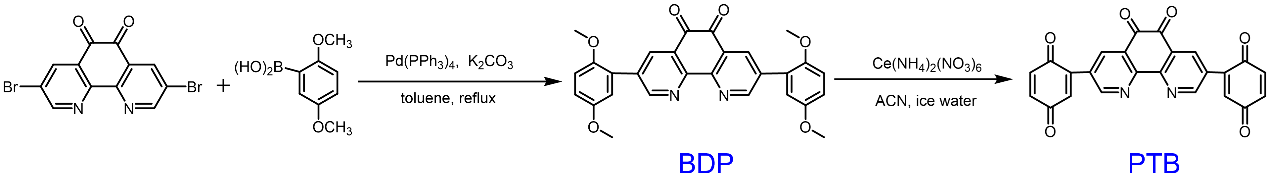
Scheme S1.** synthetic route of PTB.

**Preparation of PTB@MXSC positive electrode**

MXene (Ti₃C₂T_x_) was synthesized by etching Ti₃AlC₂ (MAX) phase in a mixture of HCl and LiF. MXene nanosheets were further bonded with carboxylated SWCNTs to make 1D-2D hierachical structure as flexible conductive substrate by following the our early reported procedure.^2^ The PTB was incorporated into MXene-SWCNTs dispersion in DI water with varied mass concentrations, followed by ultrasonication in an ice bath for 6 h to obtain a homogeneous hybrid dispersion. The resulting mixture was then vacuum-filtered and dried for 12 h to yield free-standing PTB@MXSC films. By adjusting the mass ratios of PTB, MXene and SWCNTs, flexible films with different PTB ratios were successfully fabricated. And the composites were thus designated according to weight ratio of three components, e.g. PTB@MXSC (2:7:8) has a weight ratio of 2:7:8 for PTB and MXene and SWCNTs. The obtained electrodes had a mass loading of active material in the range of 0.8–1.8 mg cm^-2^. In this work, except otherwise specified, PTB@MXSC is designated to refer to the composite with a weight ratio of 1:7:8 for PTB and MXene and SWCNTs.

**Preparation of PA@MXSC and BQ@MXSC positive electrodes**

For comparison study of organic molecules, PA@MXSC and BQ@MXSC positive electrodes were fabricated following a procedure similar to that for PTB@MXSC. In both cases, the organic molecules (PA or BQ) were uniformly mixed with MXene and SWCNTs at a fixed weight ratio of 1:7:8 (organic molecule:MXene:SWCNTs). The resulting hybrid dispersions were processed under identical conditions to obtain free-standing composite films.

**Preparation of PTB@CP positive electrode**

For comparison study of conductive carbon and binder, the PTB@CP electrode was prepared by thoroughly mixing PTB (60 wt%), carbon black (30 wt%), and polytetrafluoroethylene (PTFE, 10 wt%) in deionized water to form a homogeneous slurry and rolled into a thin membrane. After drying overnight in an oven at 60 °C, the membrane was pressed onto a stainless-steel mesh and cut into 12-mm-diameter disks. The obtained electrodes exhibited an active material mass loading in the range of 1.5-2.0 mg cm^–2^.

**Characterizations**

Nuclear magnetic resonance (NMR) spectra were recorded at 298 K on a 500 MHz Bruker superconducting magnet highfield ^1^H NMR spectrometer (500 MHz) with DMSO-*d*_6_ as the deturated solvent and ^13^C NMR spectrometer (125 MHz) with CF_3_COOD as the deturated solvent. The X-Ray diffraction (XRD) patterns were recorded by Bruker (Cu Kα, λ=0.154 nm) with a scan rate of 10° min^-1^ from 5° to 90°. Fourier transform infrared spectroscopy (FTIR, Bruker Vertex 70V) was carried out to analyze the chemical structures. The microstructure, morphology, and elemental distribution of electrodes were examined using a field-emission scanning electron microscope (GeminiSEM 500). Transmission electron microscope (TEM) images of MXene and PTB@MXSC were captured using TEM (JEOL JEM-F200). The stacking of MXene was observed using an atomic force microscope (AFM, Oxford Instruments Asylum Research Cypher ES). X-ray photoelectron spectroscopy (XPS) analysis was conducted on a Thermo FisherESCALAB XI+ photoelectron spectrometer. The composition of the powder was determined by using a matrix-assisted laser desorption/ionization time-of-flight mass spectrometer (autoflex maX MALDI-TOF MS). The specific surface areas of PTB and PTB@MXSC were obtained using an accelerated surface area and porosity measurement system (TriStar II 3020). A smooth and flat PTB@MXSC section was obtained using a triple ion beam cutter (Leica EM TIC 3X).

**Electrochemical measurements**

The electrochemical performance of the organic positive electrodes was measured with CR2032-type coin cells at room temperature. The coin cells were assembled using organic positive electrodes, glass fiber separator (Whatman GF/D), 4 M Zn(ClO_4_)_2_ aqueous electrolyte, and Zn foil negative electrode (thickness: 100 µm; diameter: 15 mm; Purity: ≥99.9%) in the atmospheric environment. Unless otherwise stated, 4 M Zn(ClO₄)₂ aqueous solution was used as the electrolyte for all cells in this work. Cyclic voltammetry (CV) curves and electrochemical impedance spectroscopy (EIS) were collected on a multi-functional electrochemical workstation (CHI 760E, Chenhua). All galvanostatic charge/discharge (GCD) measurements were conducted by using a multichannel NEWARE BTS-800 battery test system.

**Calculation of theoretical specific capacity**

The theoretical specific capacity of the PA, BQ and PTB can be calculated according to the following equation:

$$Q_{spec}=\frac{n\times F}{3.6\times M_{w}}$$

Where *Q_spec_* is the theoretical specific capacity (mAh g^-1^), n is the theoretical electron-transfer numbers of PA (2-electron reaction), BQ (2-electron reaction) and PTB (6-electron reaction), F is the Faraday constant (96485 C mol^-1^) and M_w_ is the relative molecular mass of the PA (180.21 g mol^-1^), BQ (108.09 g mol^-1^) and PTB (392.37 g mol^-1^). As a result, the theoretical specific capacities of the PA, BQ and PTB are calculated to be about 297.45 mAh g^-1^, 495.91 mAh g^-1^ and 409.84 mAh g^-1^, respectively.

**Calculation of the ion diffusion coefficient by GITT**

The ion diffusion coefficient in the PTB@MXSC positive electrode is calculated based on the following equation:

$$D_{s}=\frac{4}{\pi\tau}\left( \frac{m_{B}V_{M}}{M_{B}S} \right)^{2}\left( \frac{\Delta E_{S}}{\Delta E_{\tau}} \right)^{2}$$

Where *τ* is the relaxation time, *M_B_*, *S*, *m_B_* and *V_M_* are the molar mass, electrode-electrolyte interface area, mass and molar volume of electrode material, respectively. *ΔE_S_* and *ΔE_τ_* are voltage drops.

**Theoretical calculation**

All the DFT calculations were performed by using Gaussian 16 package.^3^ The geometries optimization and frequencies calculations were computed by employing the B3LYP-D3(BJ) functional in conjunction with 6-31G(d,p) basis set.^4^ We used B3LYP functional and def2-TZVP basis set to perform single-energy calculations.^5^ The adsorption energy was evaluated according to the following formula:

$$E=E_{PTB+ion}-E_{PTB}-E_{ion}$$

where $E_{PTB+ion}$ is the total energy of the adsorbed system, $E_{PTB}$ is the energy of the optimized PTB molecular and $E_{ion}$ is the energy of Zn^2+^ or H^+^. The electrostatic potential (ESP) analysis and visualization were conducted using Multiwfn and VMD.^6,7^ Moreover, the theoretical calculations were conducted to examine the molecular orbital levels of the investigated molecule, which encompassed the highest occupied molecular orbital (HOMO) and the lowest unoccupied molecular orbital (LUMO).


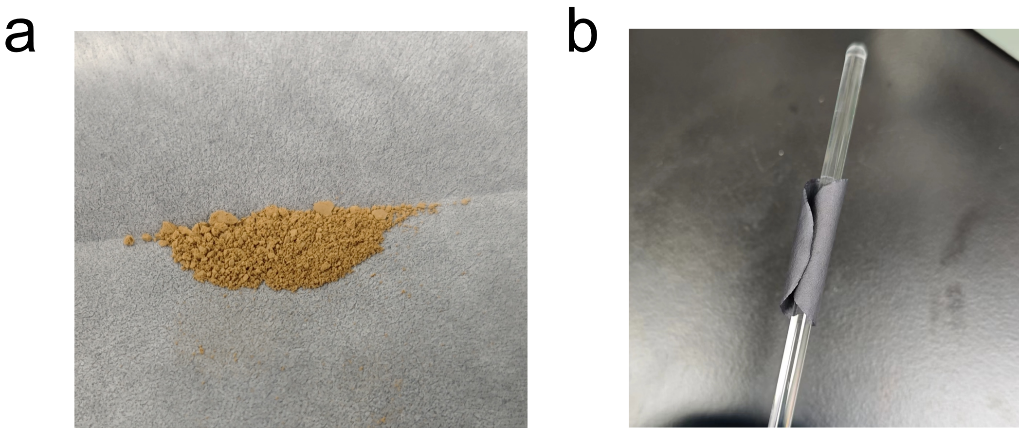


**Figure S1.** The digital images of (a) PTB and (b) PTB@MXSC.


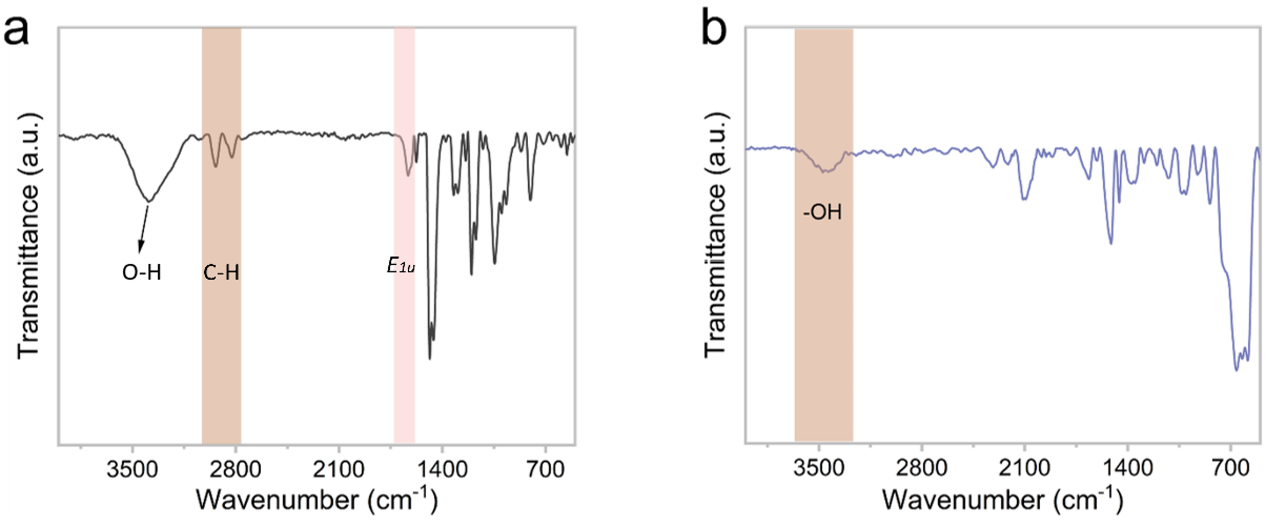


**Figure S2.** FTIR spectra of (a) Carboxyated SWCNTs and (b) MXene.


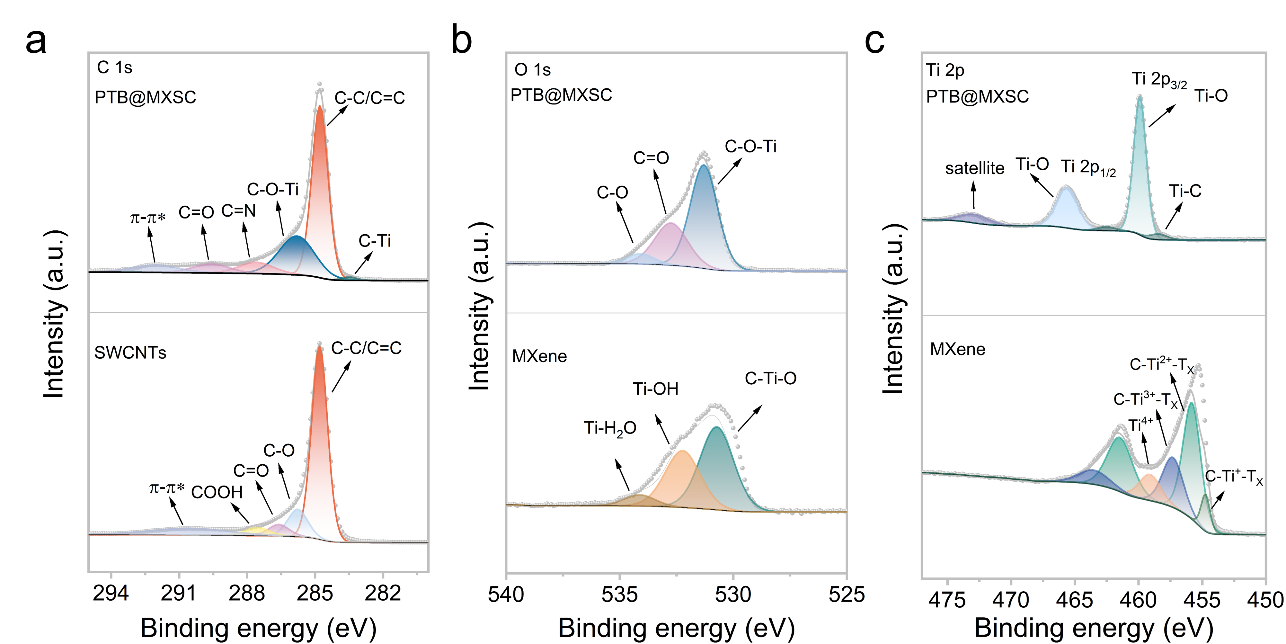


**Figure S3**. XPS spectra of (a) C 1s of PTB@MXSC and SWCNTs, (b) O 1s and (c) Ti 2p of PTB@MXSC and MXene.


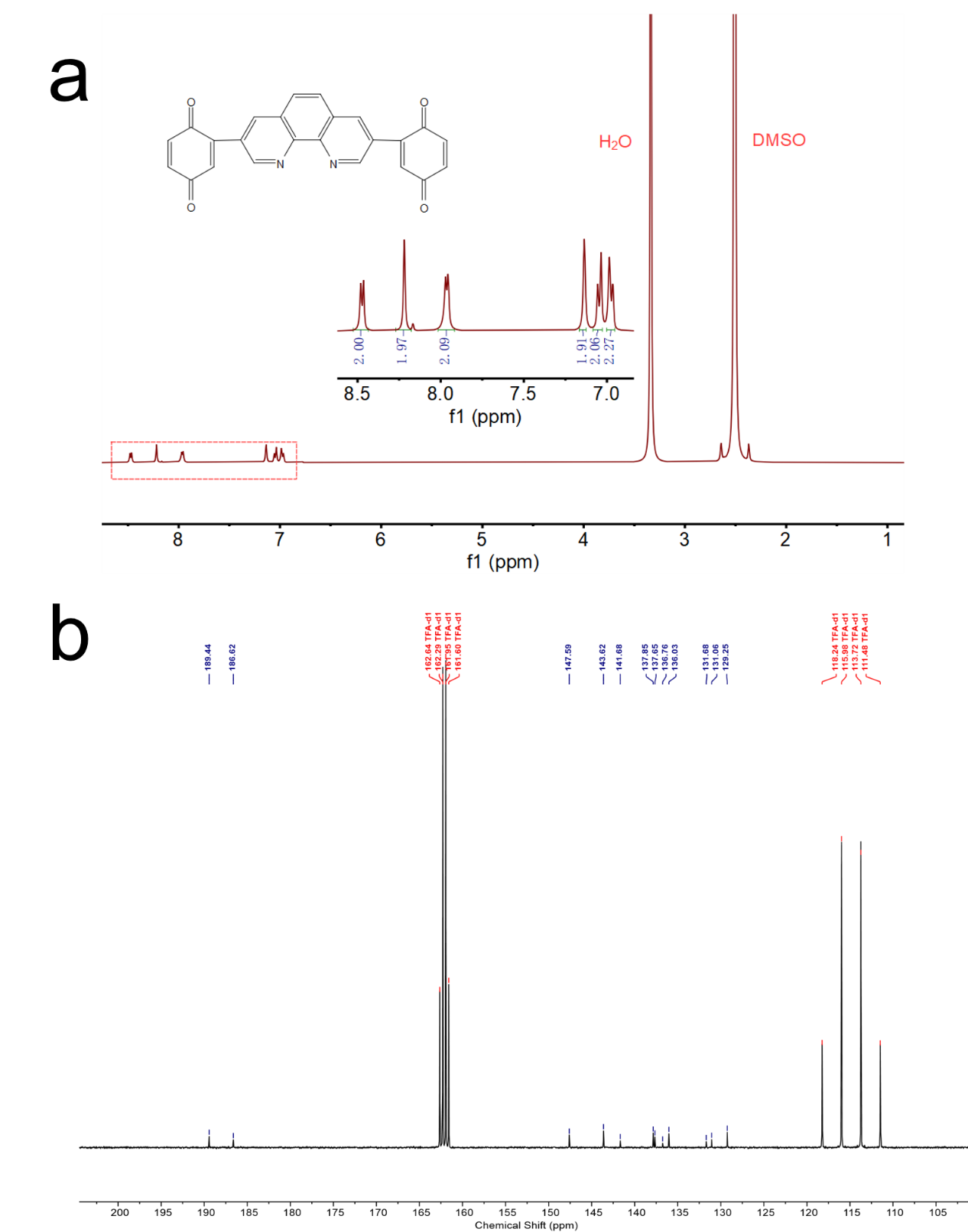


**Figure S4.** (a) ^1^H NMR (500 MHz, DMSO-*d*_6_) and (b) ^13^C NMR (125 MHZ, TFA-d) spectra of PTB.


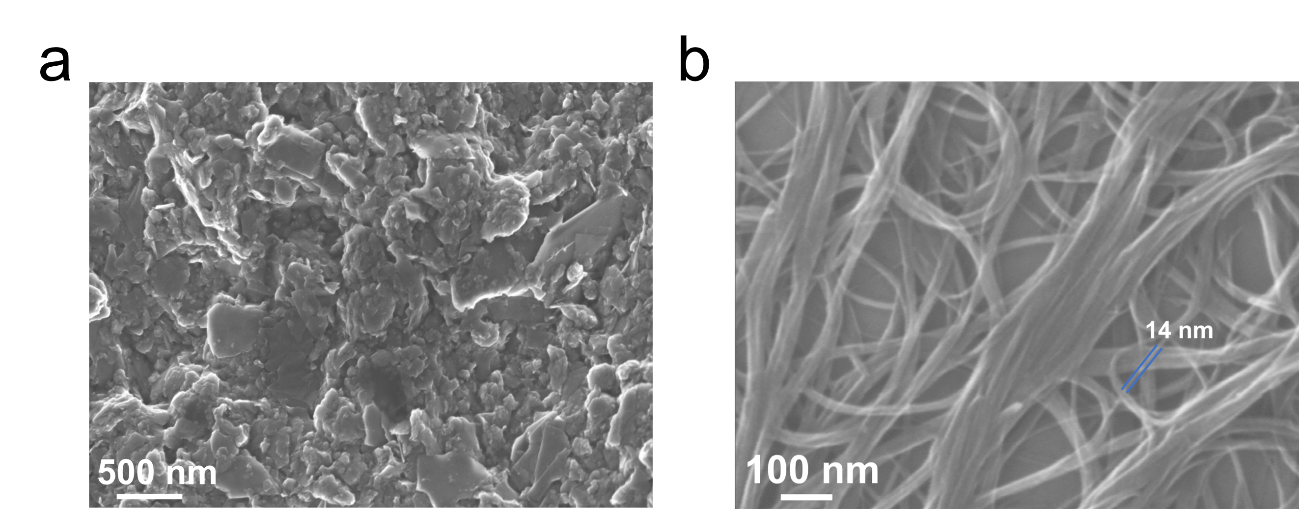


**Figure S5.** SEM images of (a) PTB and (b) SWCNTs.


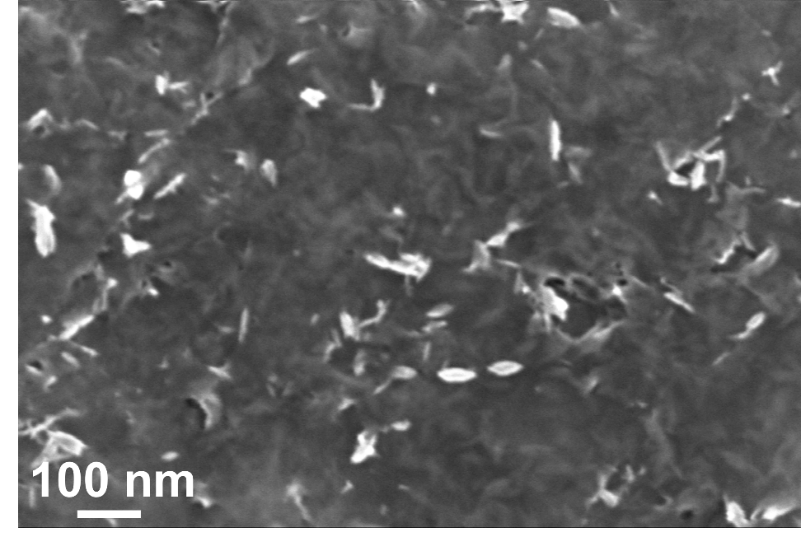


**Figure S6.** SEM image of MXene after ultrasonication.


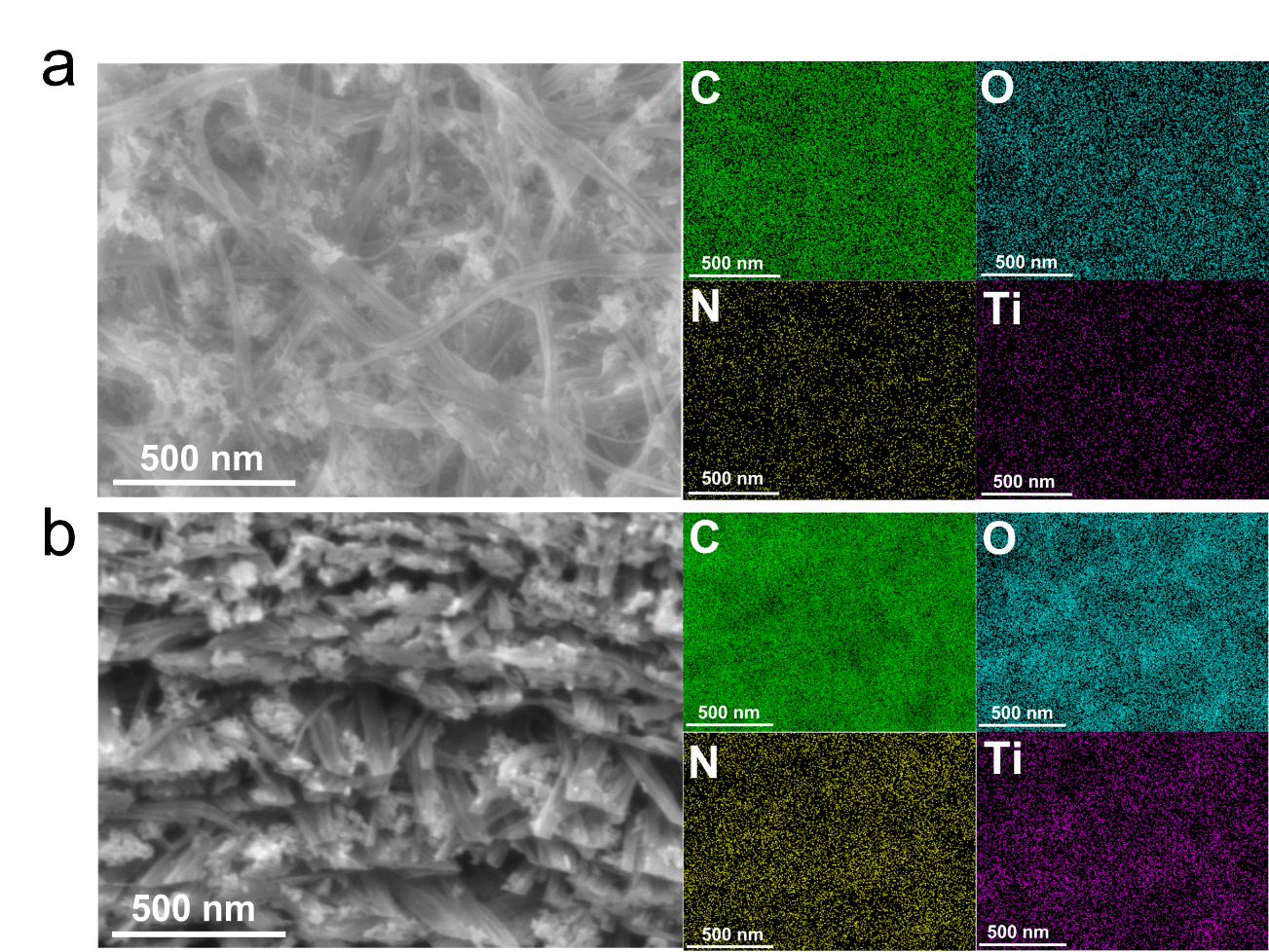


**Figure S7.** (a) Planar-view and (b) Cross-sectional SEM and EDS mapping images of the PTB@MXSC.


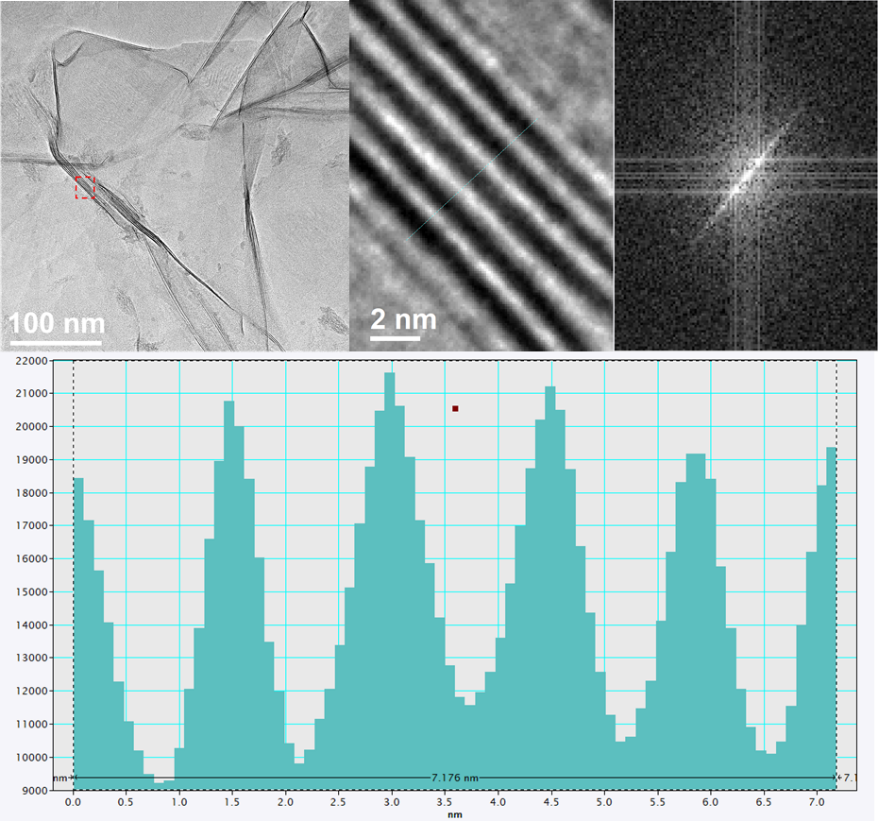


**Figure S8.** TEM images of the MXene.


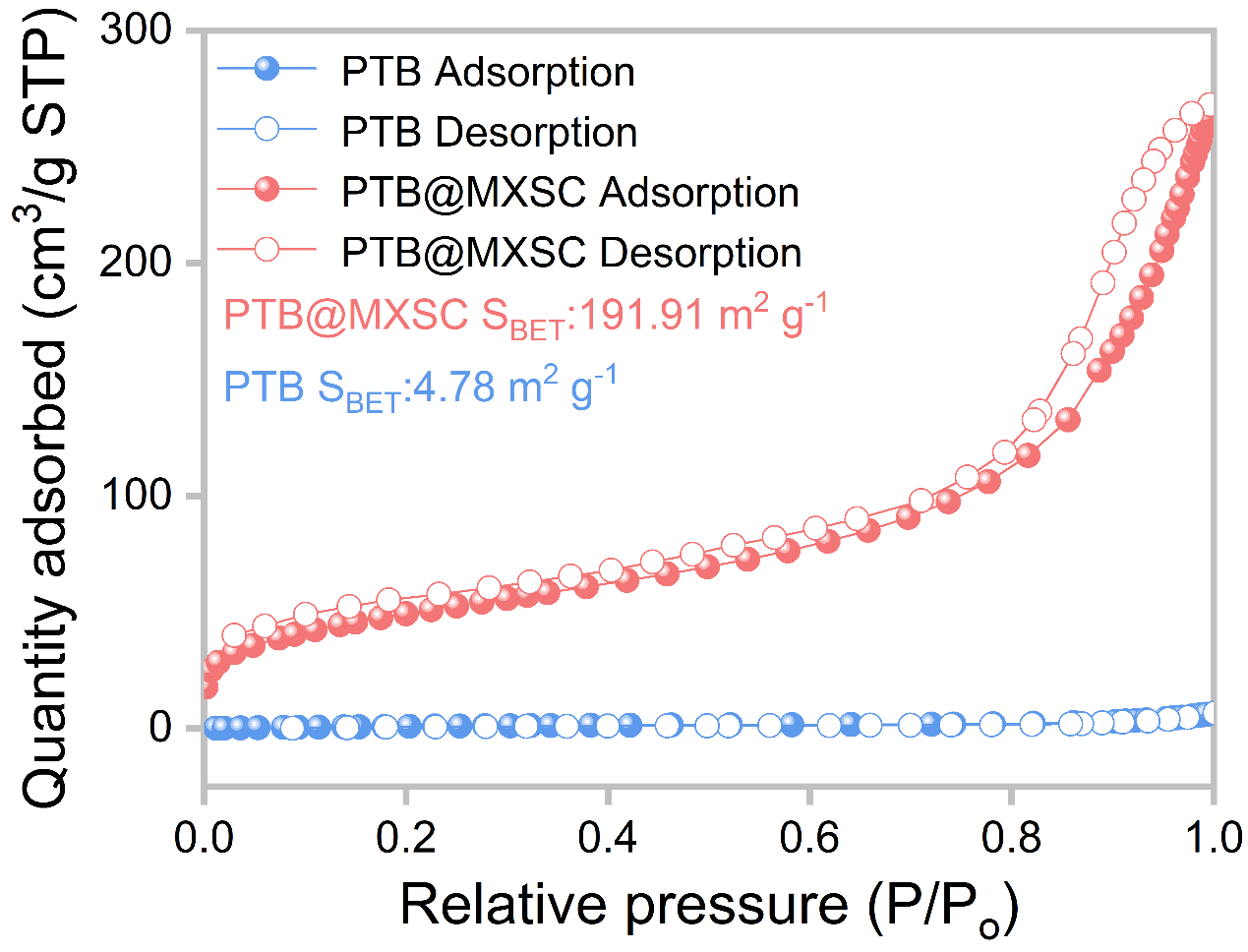


**Figure S9.** N_2_ adsorption–desorption isotherm curves of PTB and PTB@MXSC.


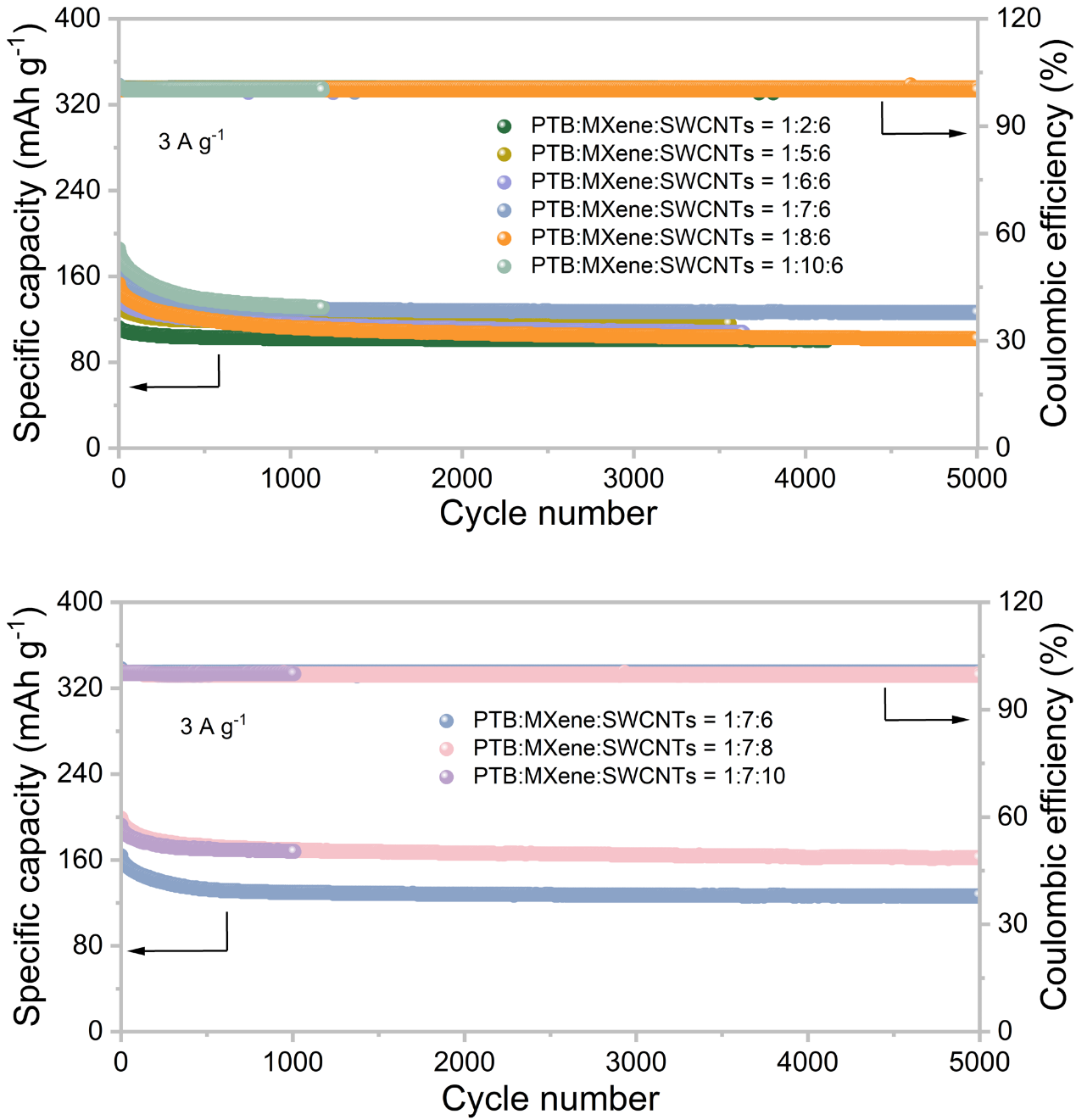


**Figure S10.** Cycling performance of PTB@MXSC with varying component ratios.


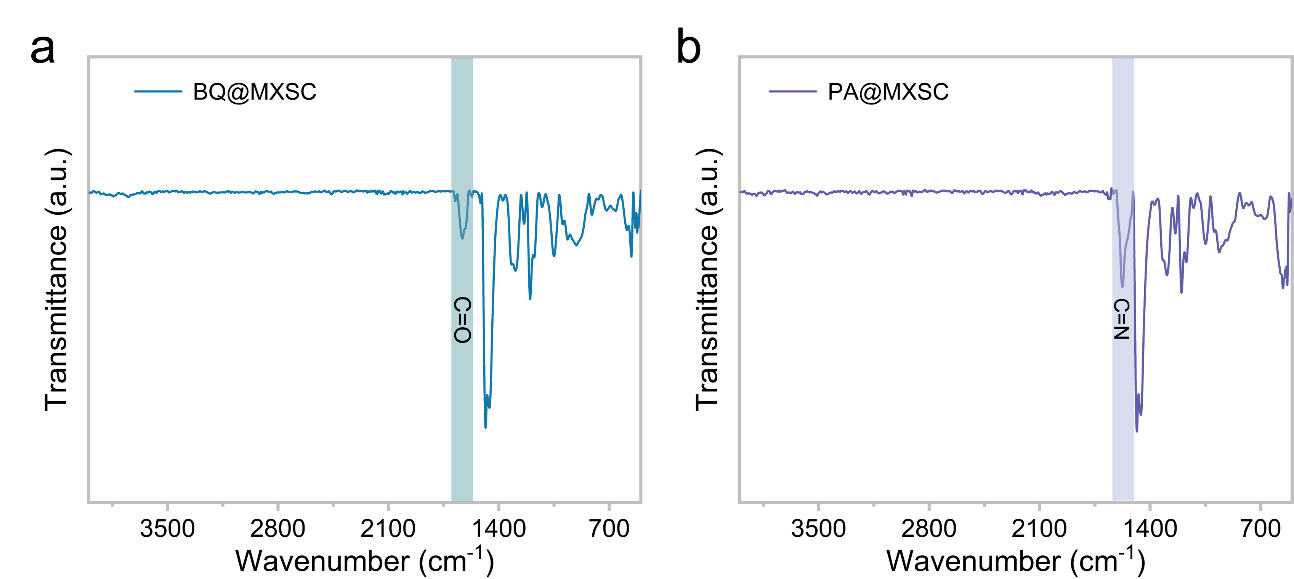


**Figure S11.** FTIR spectra of (a) PA@MXSC and (b) BQ@MXSC.


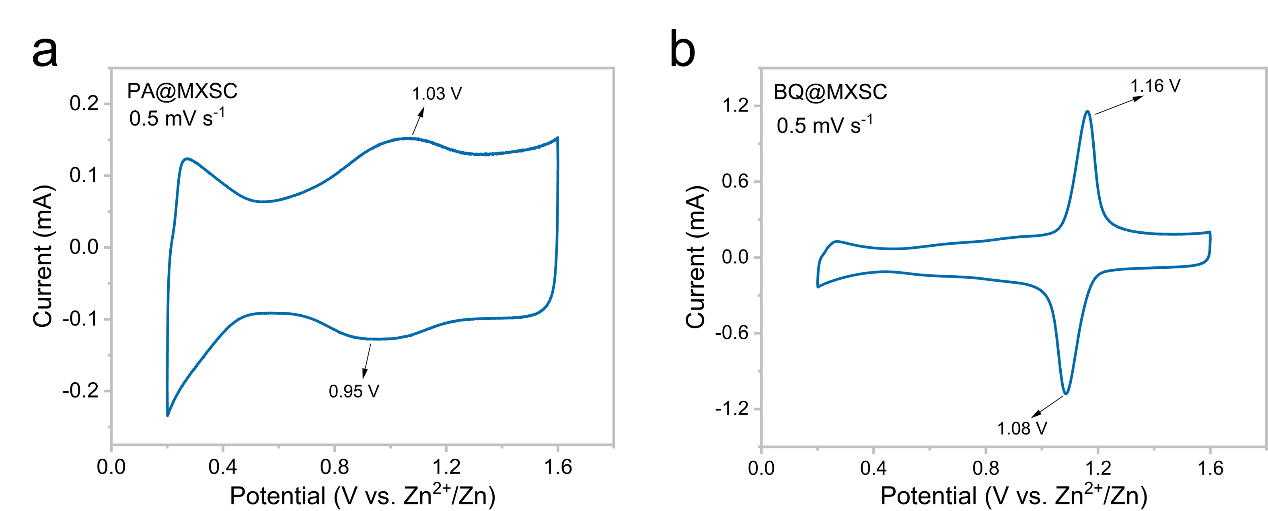


**Figure S12.** CV curves of (a) PA@MXSC and (b) BQ@MXSC positive electrodes tested at the scan rate of 0.5 mV s^-1^.


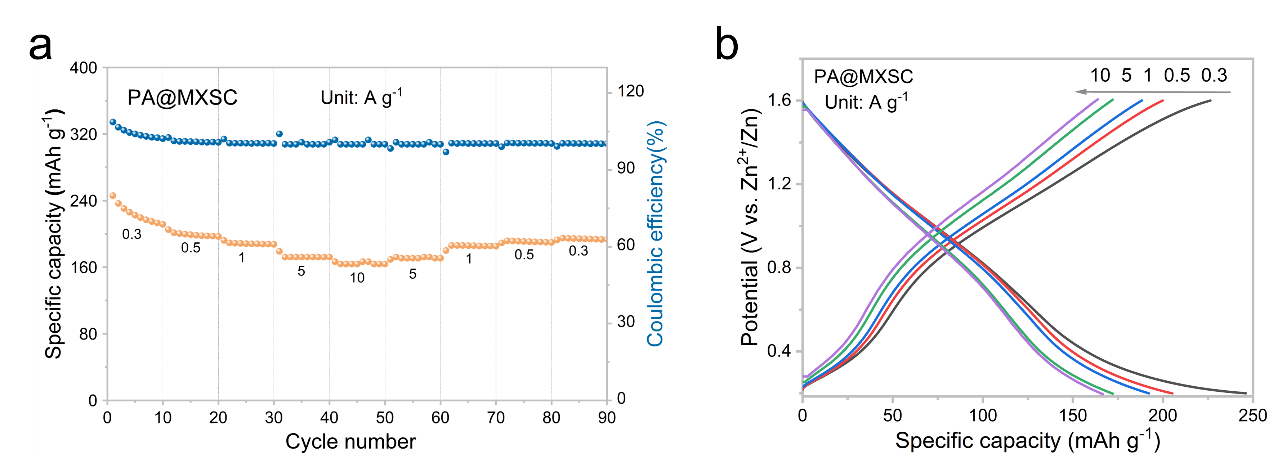


**Figure S13.** **(**a) Rate performance and (b) GCD curves of PA@MXSC in the current density range of 0.3-10 A g^-1^.


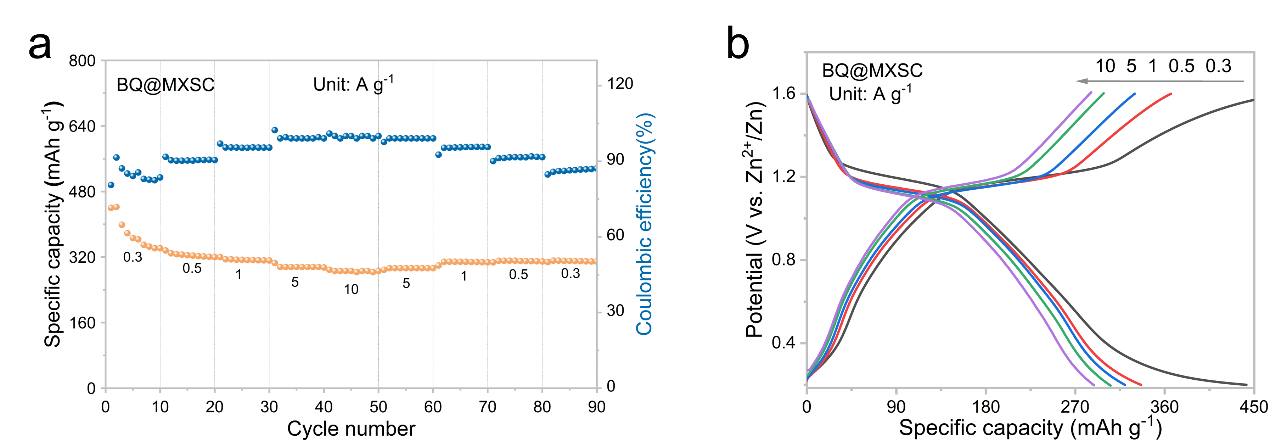


**Figure S14.** (a) Rate performance and (b) GCD curves of BQ@MXSC in the current density range of 0.3-10 A g^-1^.


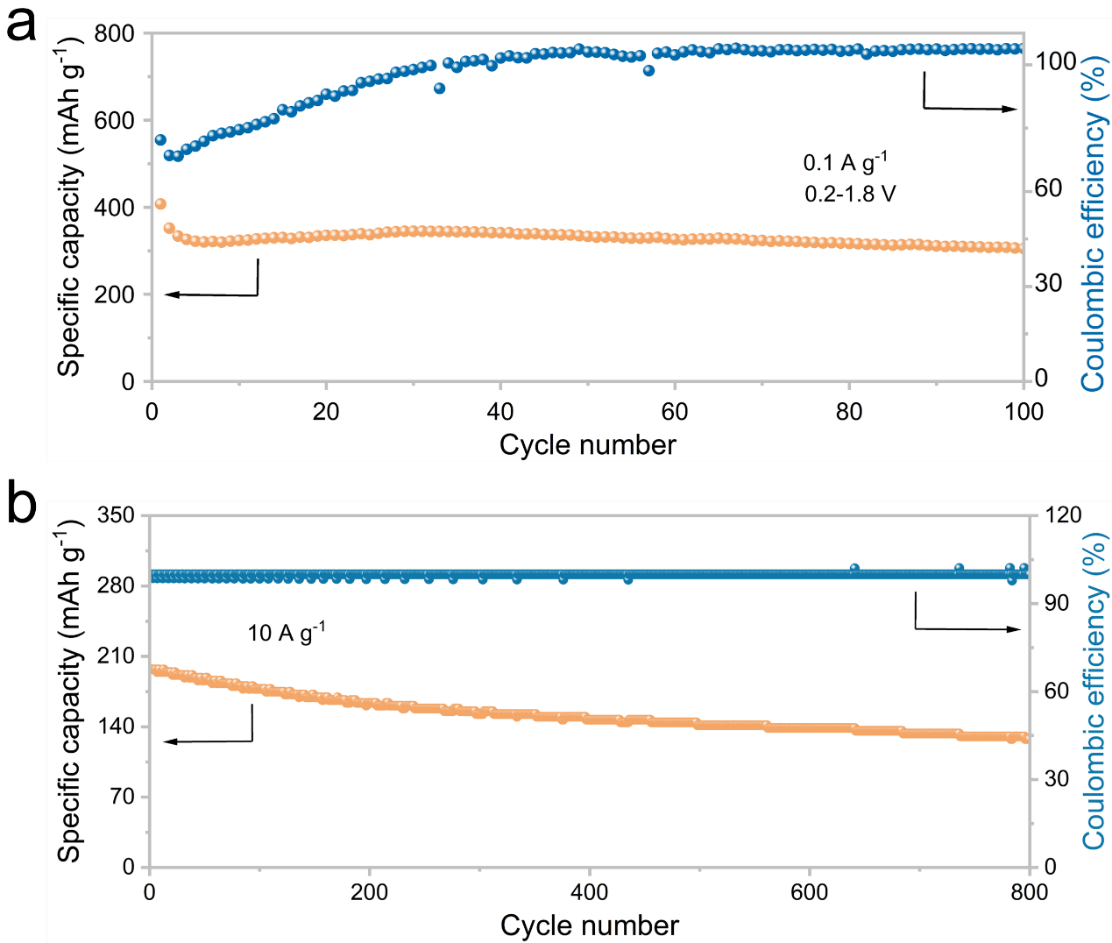


**Figure S15.** Cycling performance of PTB@MXSC at (a) 0.1A g^-1^ and (b) 10 A g^-1^ in voltage range of 0.2-1.8 V vs. Zn^2+^/Zn.


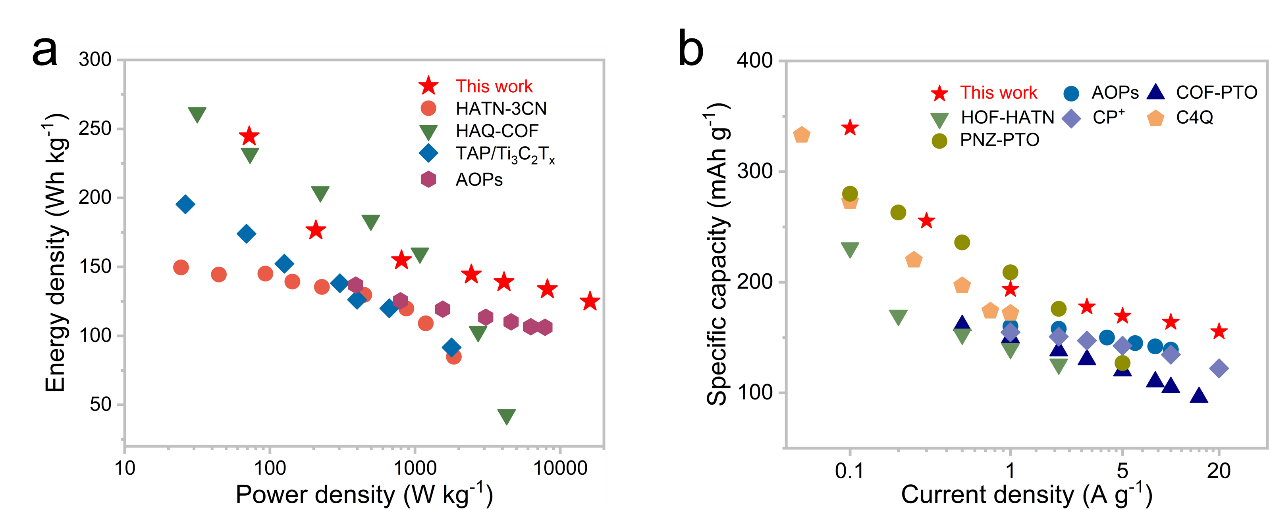


**Figure S16.** Comparison of (a) energy density versus power density and (b) rate performances between this work and previous reports on organic positive electrode materials for ZIBs.^8-16^


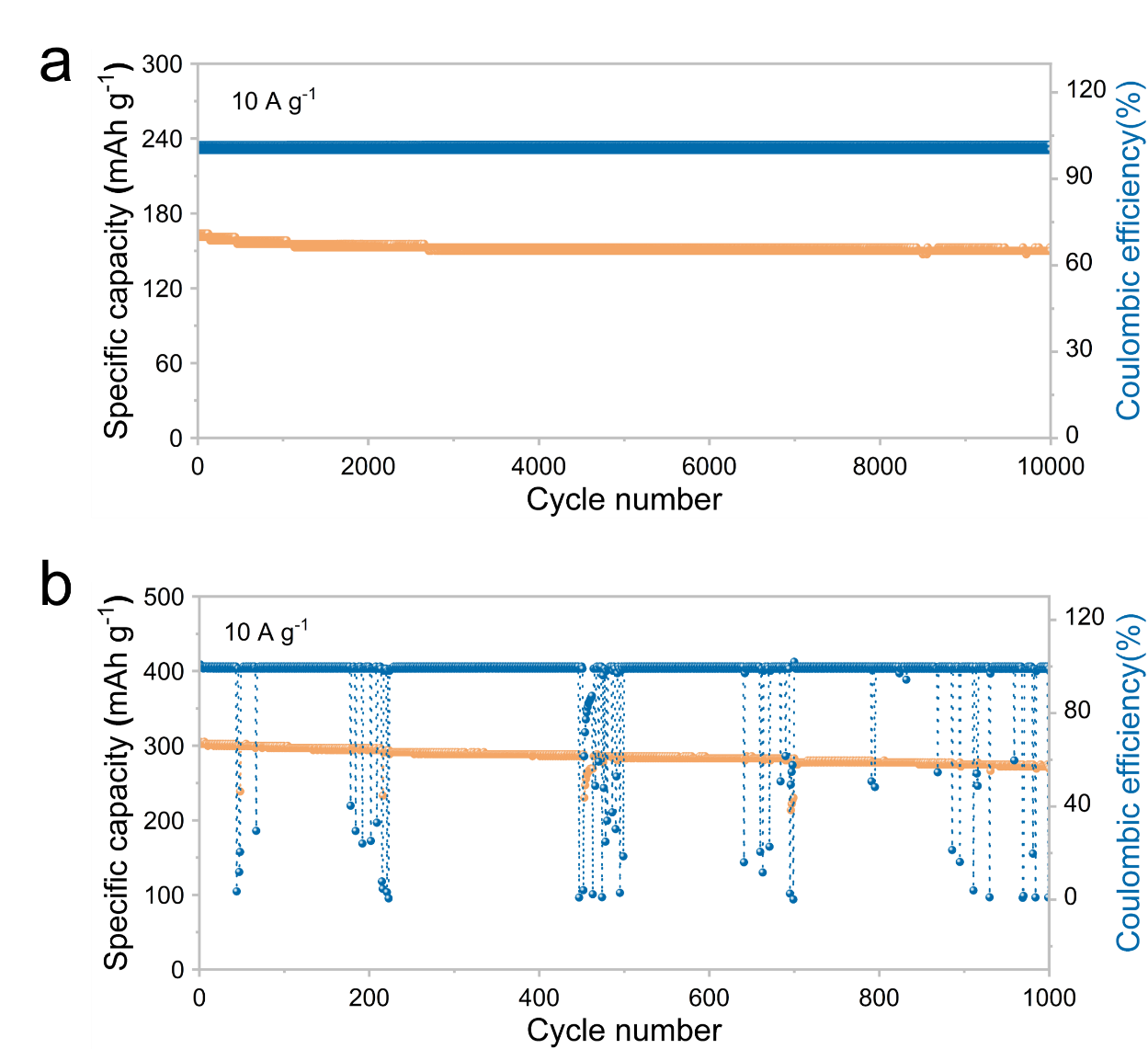


**Figure S17.** Cycling performances of (a) PA@MXSC and (b) BQ@MXSC at 10 A g^-1^.


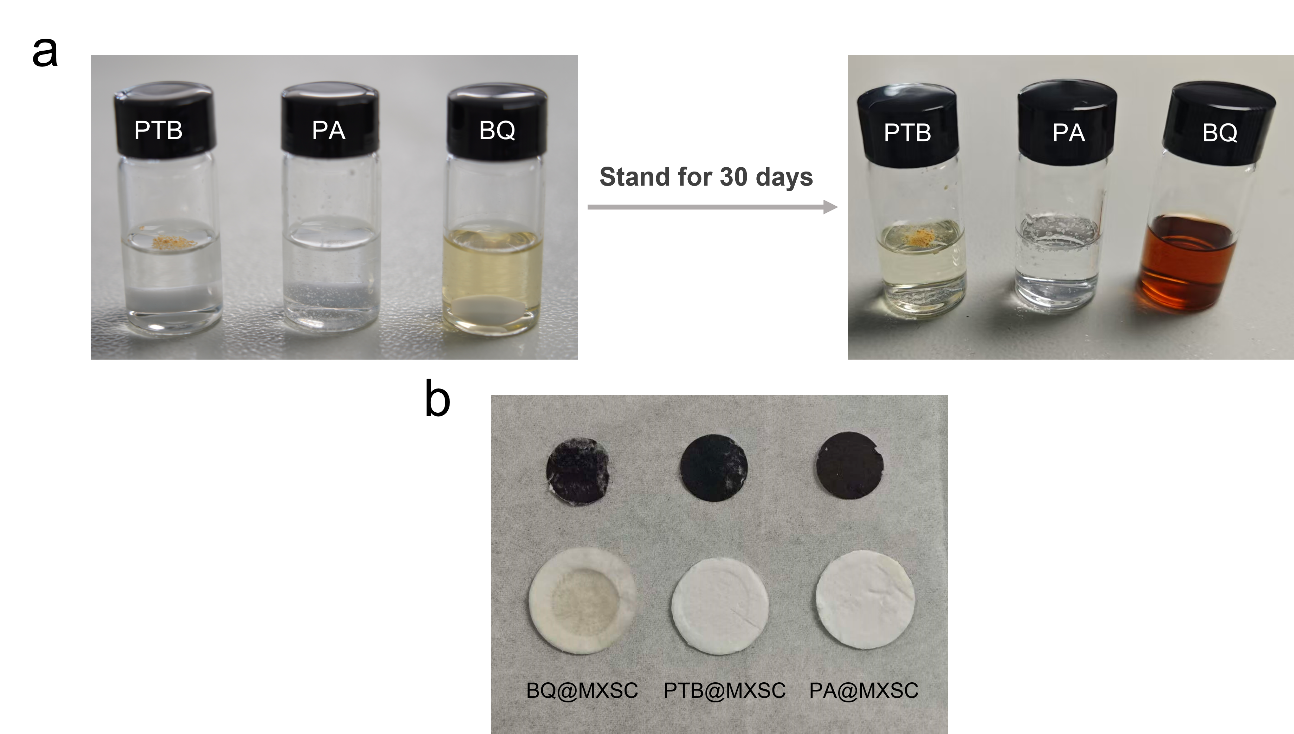


**Figure S18.** (a) The digital images of solubility characteristics of the PTB, PA and BQ in the electrolyte, and (b) Dissolution of cathodes after 1000 cycles at 10 A g^-1^.


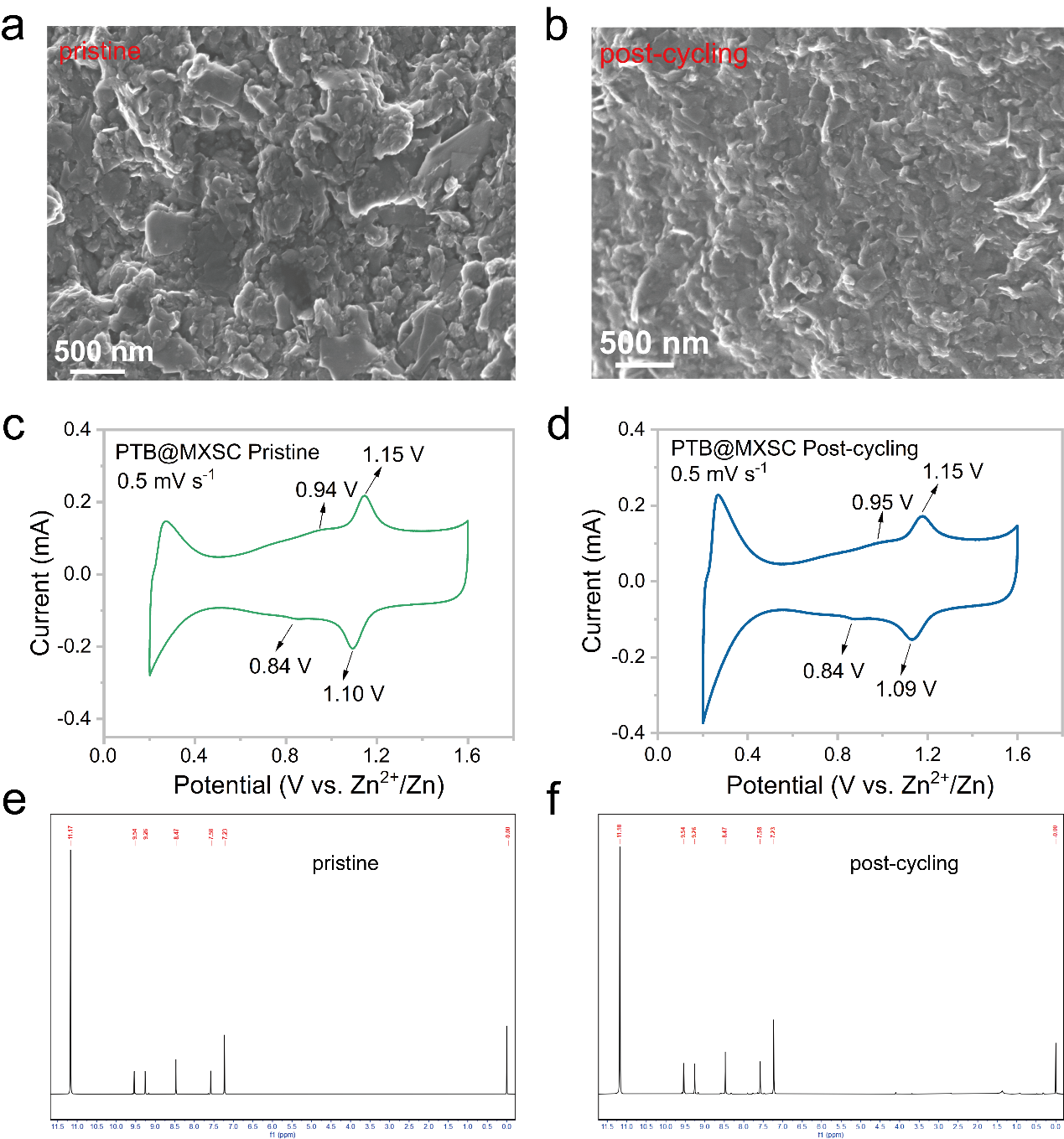


**Figure S19**. Comprehensive characterization of the PTB before and after long-term cycling. SEM images of PTB (a) before and (b) after 1000 cycles at 3 A g⁻¹. CV curves of PTB@MXSC cathode (c) before and (d) after 1000 cycles at 3 A g⁻¹. (e) ¹H NMR spectrum (500 MHz, TFA-d) of the as-synthesized PTB molecule. (f) ¹H NMR spectrum of the PTB molecule recovered from the electrode after 1000 cycles.


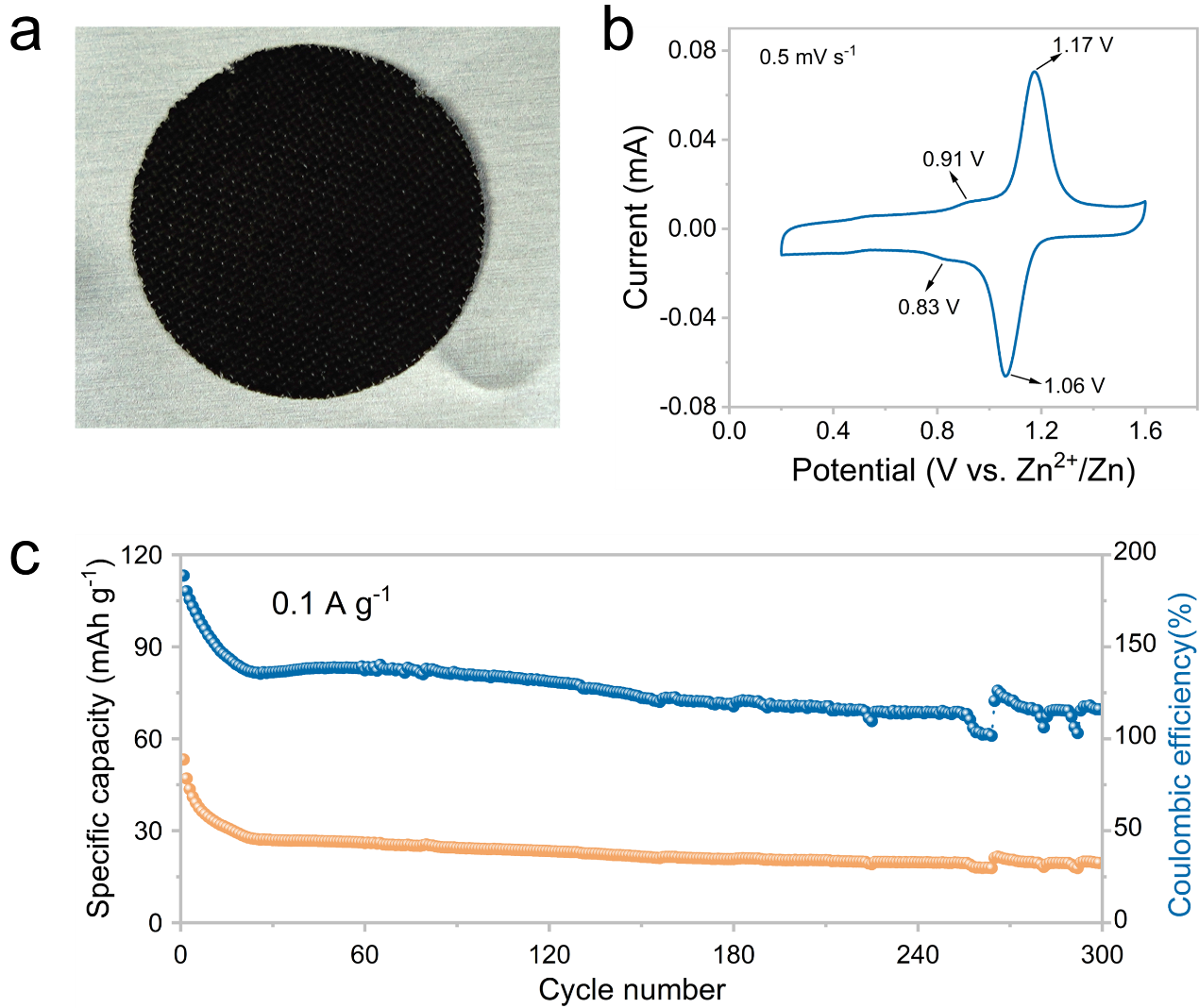


**Figure S20.** (a) The digital image and (b) CV curve and (c) Cycling performance of PTB@CP positive electrode.


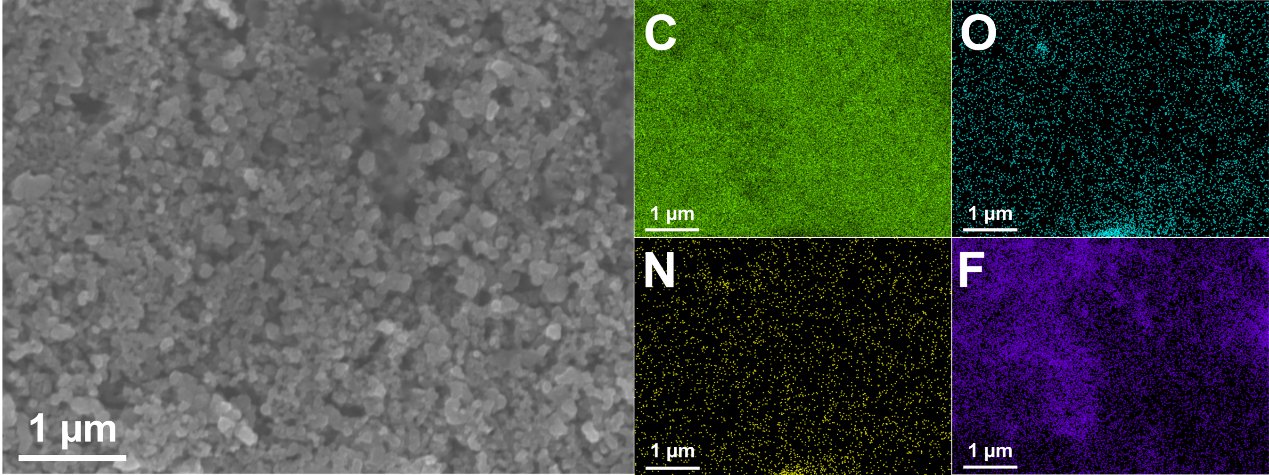


**Figure S21.** SEM and EDS mapping images of the PTB@CP.


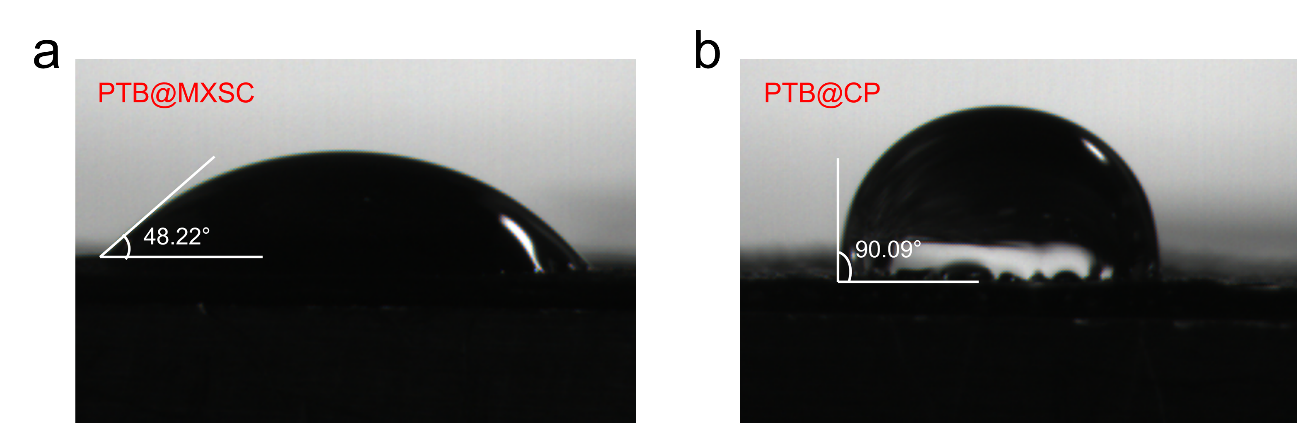


**Figure S22.** Contact angle of the 4 M Zn(ClO_4_)_2_ electrolyte with the (a) PTB@MXSC and (b) PTB@CP electrode plates.


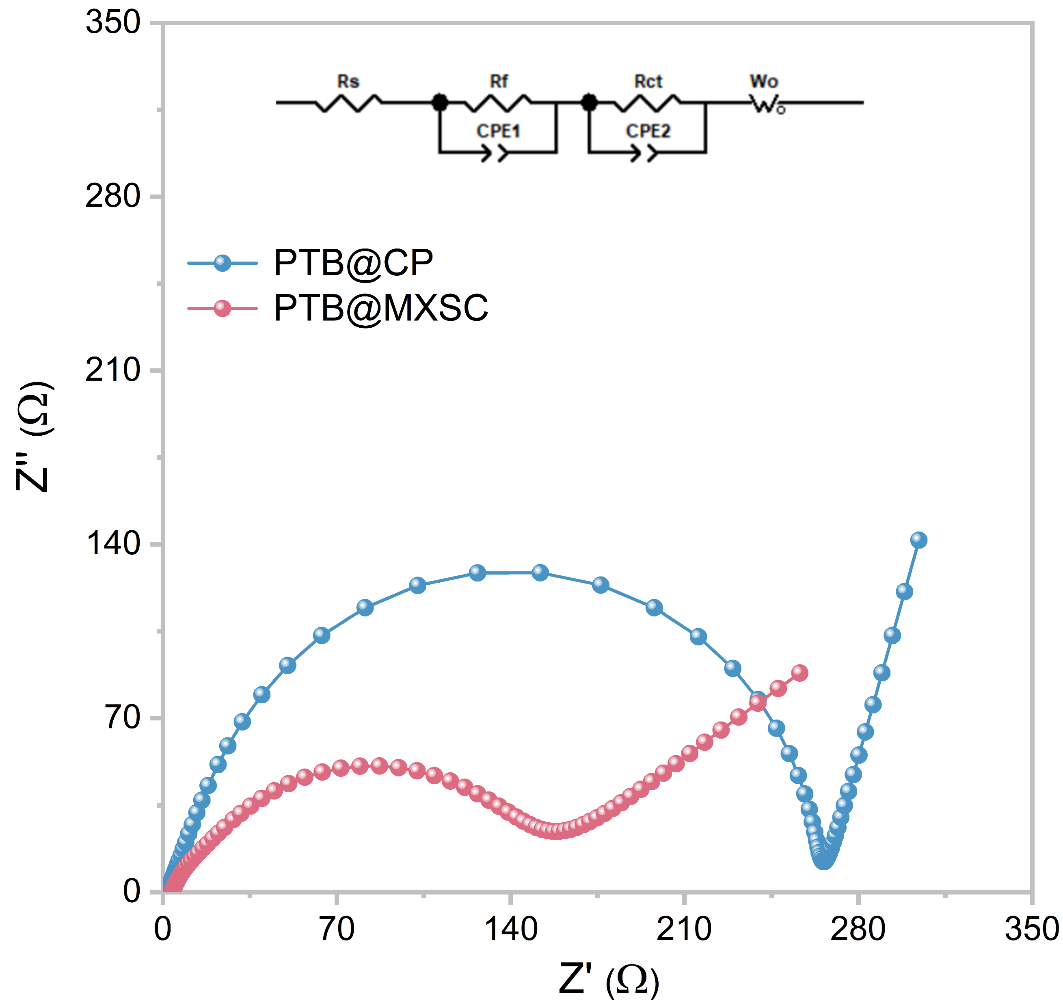


**Figure S23.** Nyquist plots of the PTB@CP//Zn and PTB@MXSC//Zn cells, the inset figure is the equivalent circuit.

**Table S1**. EIS fitting results of the PTB@CP//Zn and PTB@MXSC//Zn cells after 10^th^ cycle at 3 A g^-1^.

| parameter | PTB@CP//Zn | PTB@MXSC//Zn |
| --- | --- | --- |
| R_s_/Ω | 4.02 | 3.58 |
| R_f_/Ω | 51.69 | 8.4 |
| R_ct_/Ω | 211.9 | 137 |
| W_o_-R/Ω | 285.3 | 392.4 |


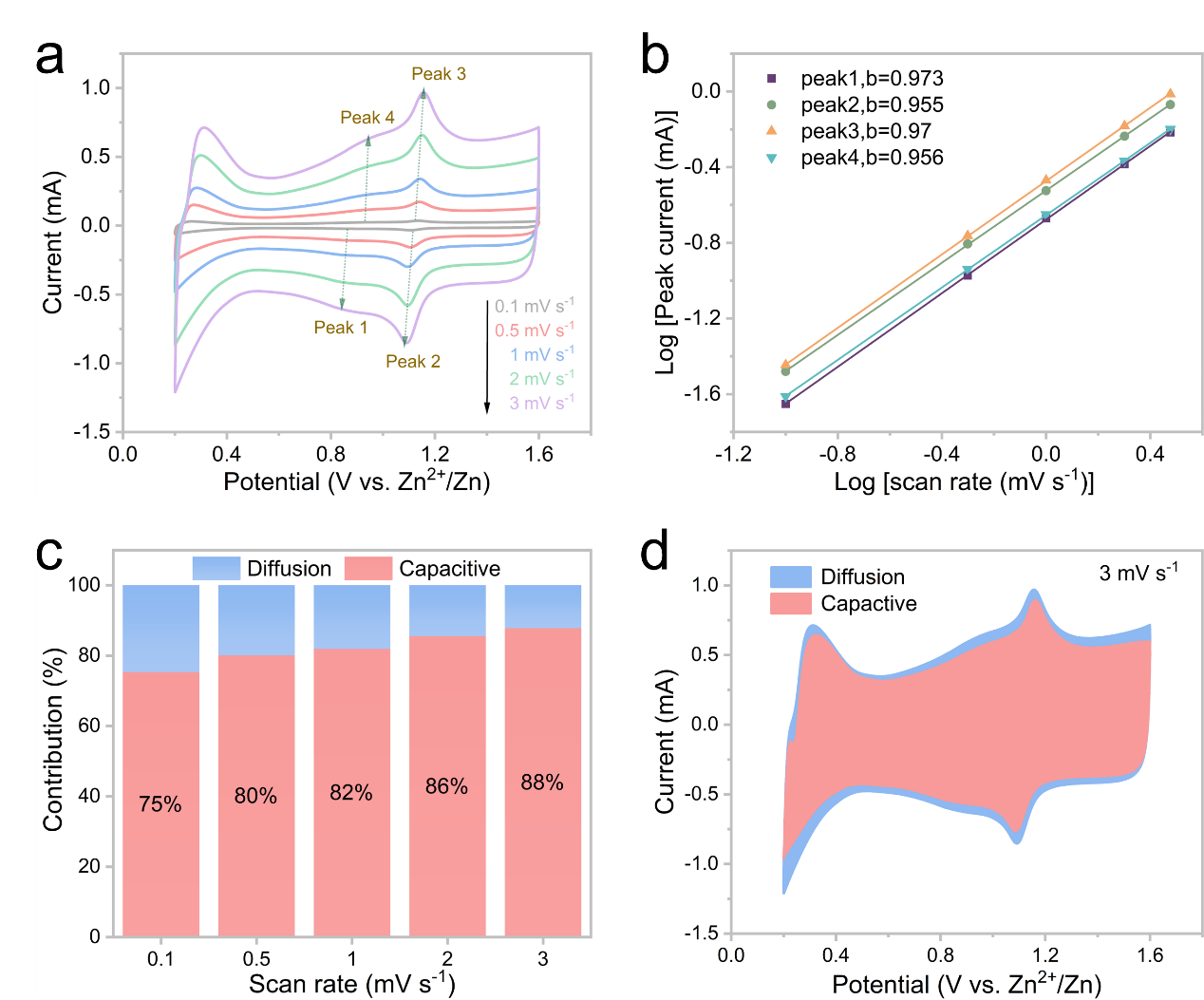


**Figure S24.** Reaction kinetic analysis of PTB@MXSC in 4 M Zn(ClO_4_)_2_. (a) CV curves at different scan rates. (b) Power law dependence of measured current on scan rate at corresponding peak potentials. (c) Contribution ratios of the capacitive charge storage at different scan rates. (d) Capacitive contribution at 3 mV s^-1^.


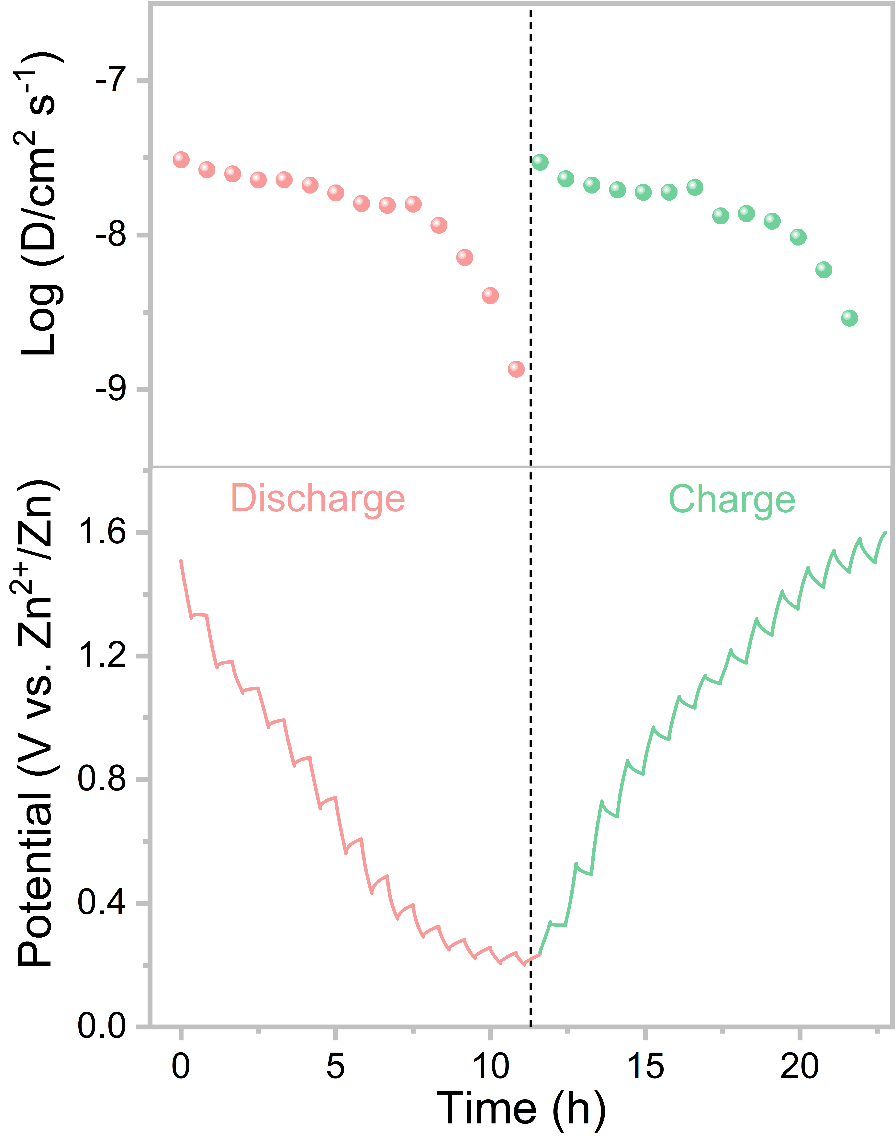


**Figure S2****5.** GITT curves and calculated chemical diffusion coefficients of PTB@MXSC.


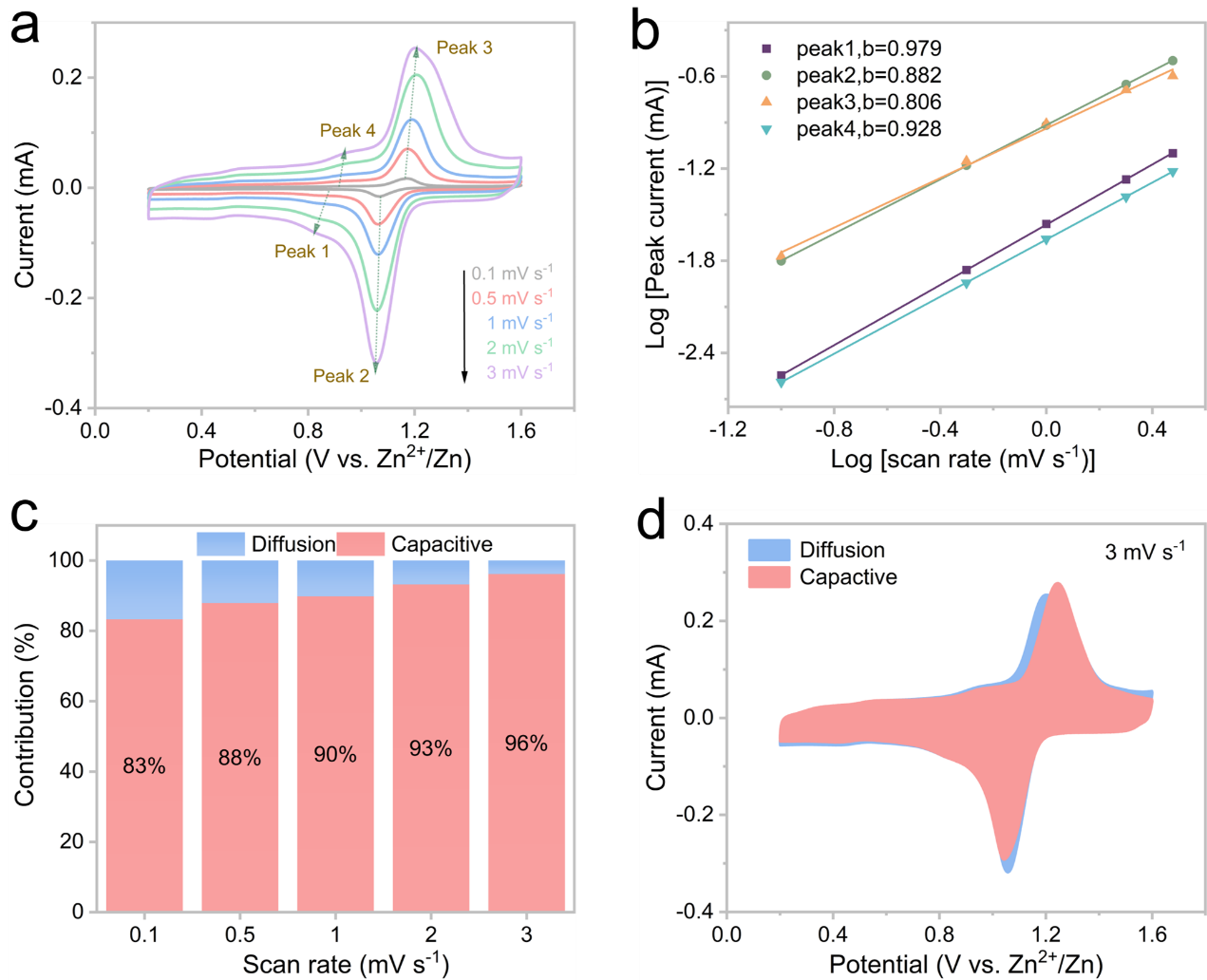


**Figure S26.** Reaction kinetic analysis of PTB@CP in 4 M Zn(ClO_4_)_2_. (a) CV curves at different scan rates. (b) Power law dependence of measured current on scan rate at corresponding peak potentials. (c) Contribution ratios of the capacitive charge storage at different scan rates. (d) Capacitive contribution at 3 mV s^-1^.


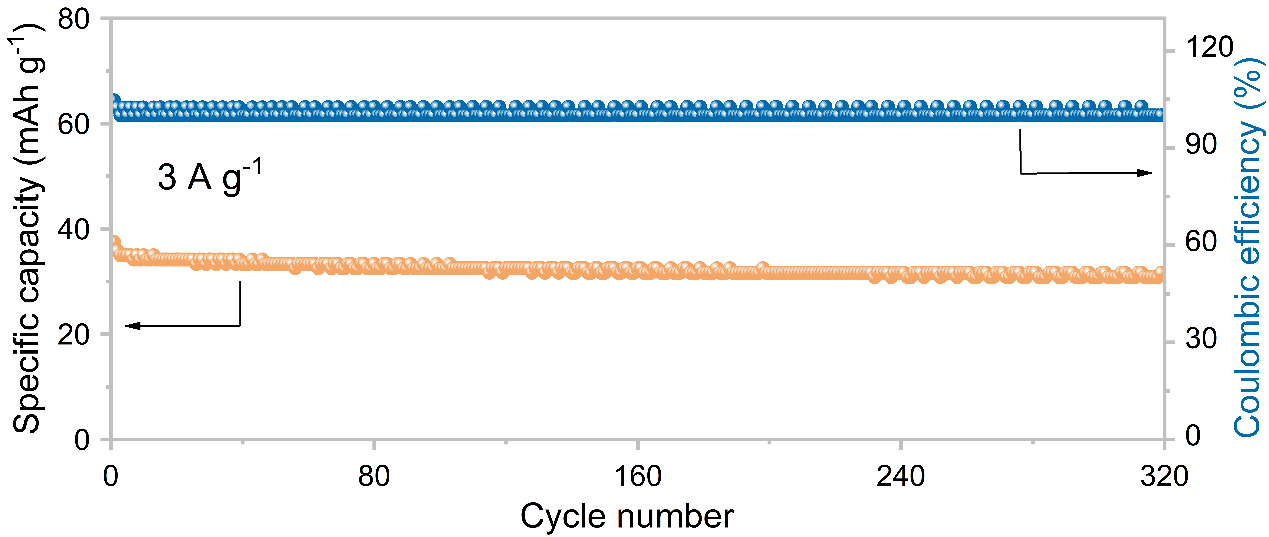


**Figure S27.** Cycling performance of MXSC.


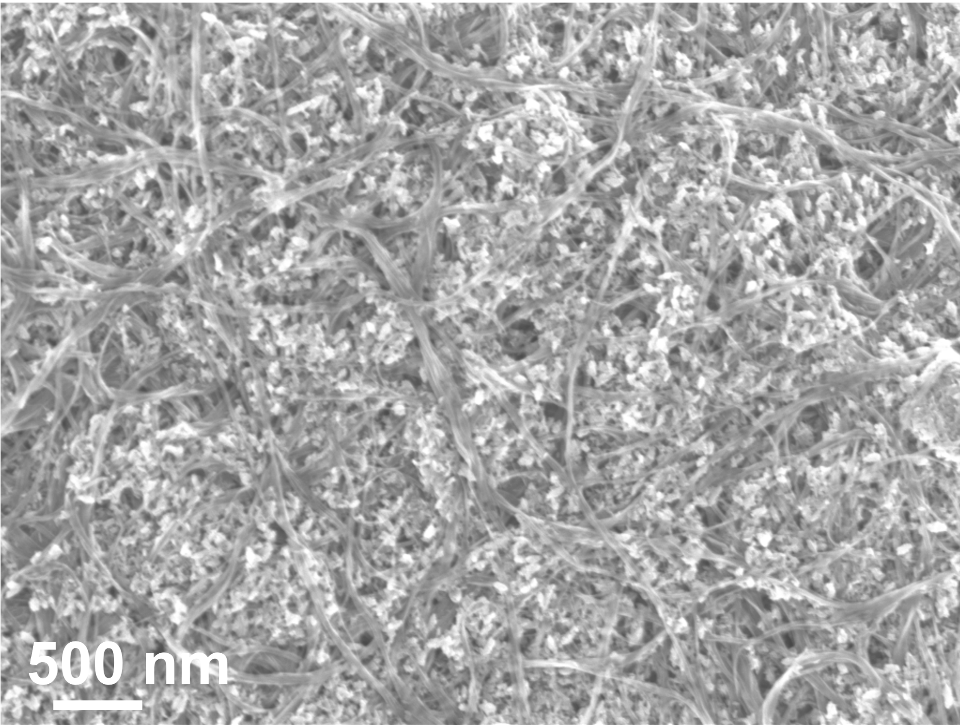


**Figure S28.** SEM image of PTB@MXSC (2:7:8).


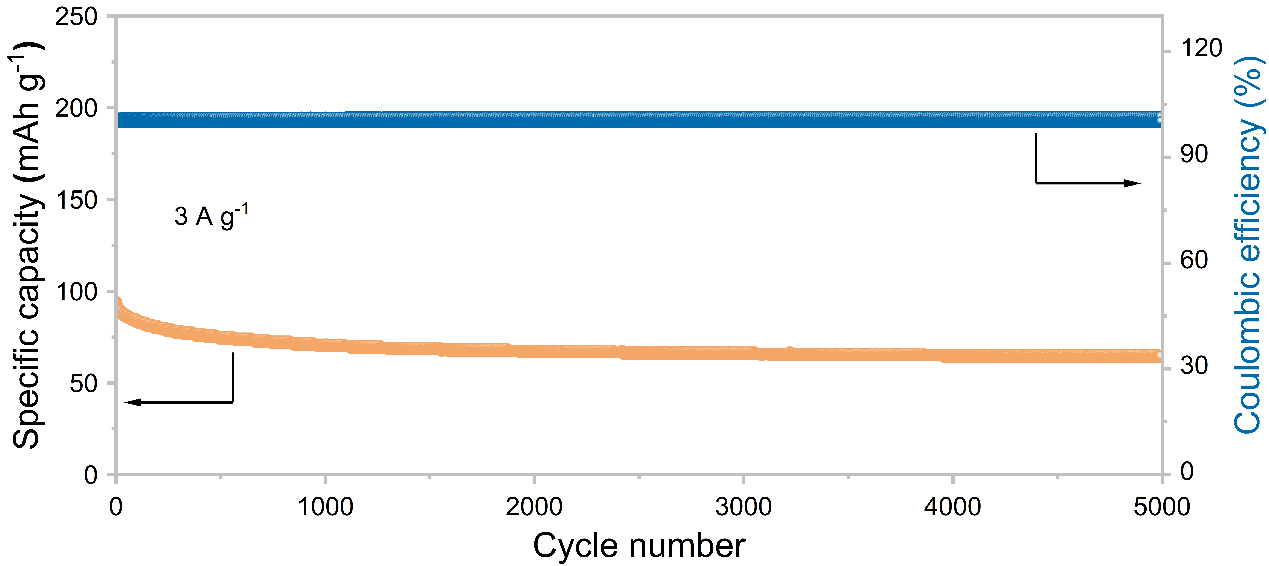


**Figure S29.** Cycling performance of PTB@MXSC (2:7:8).


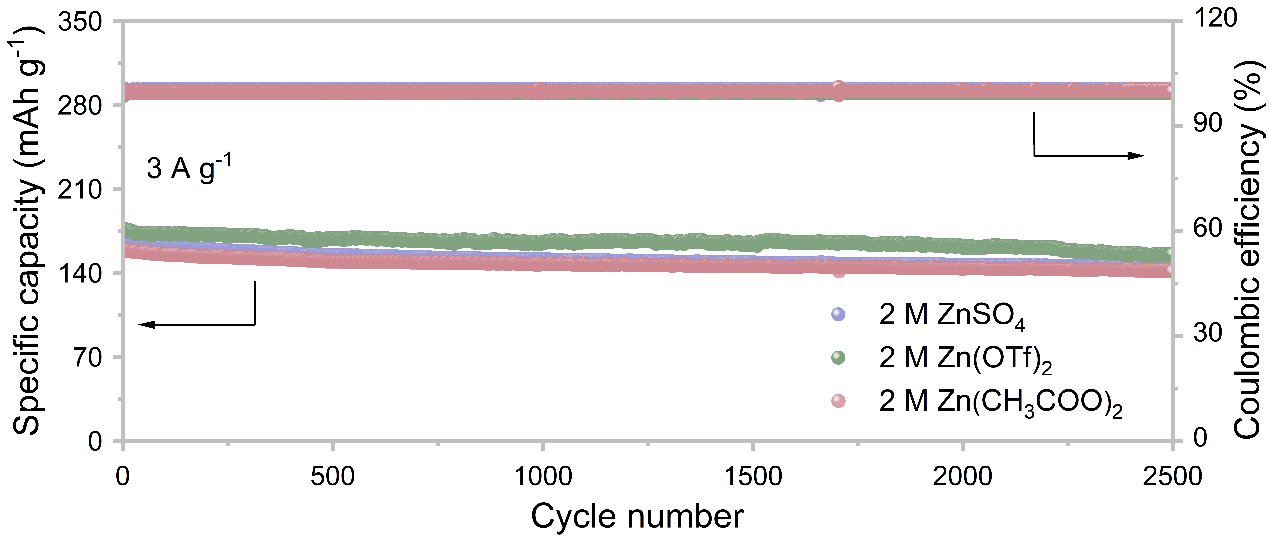


**Figure S30.** Cycling performances of PTB@MXSC in 2 M ZnSO_4_, 2 M Zn(OTf)_2_ and 2 M Zn(CH_3_COO)_2_.


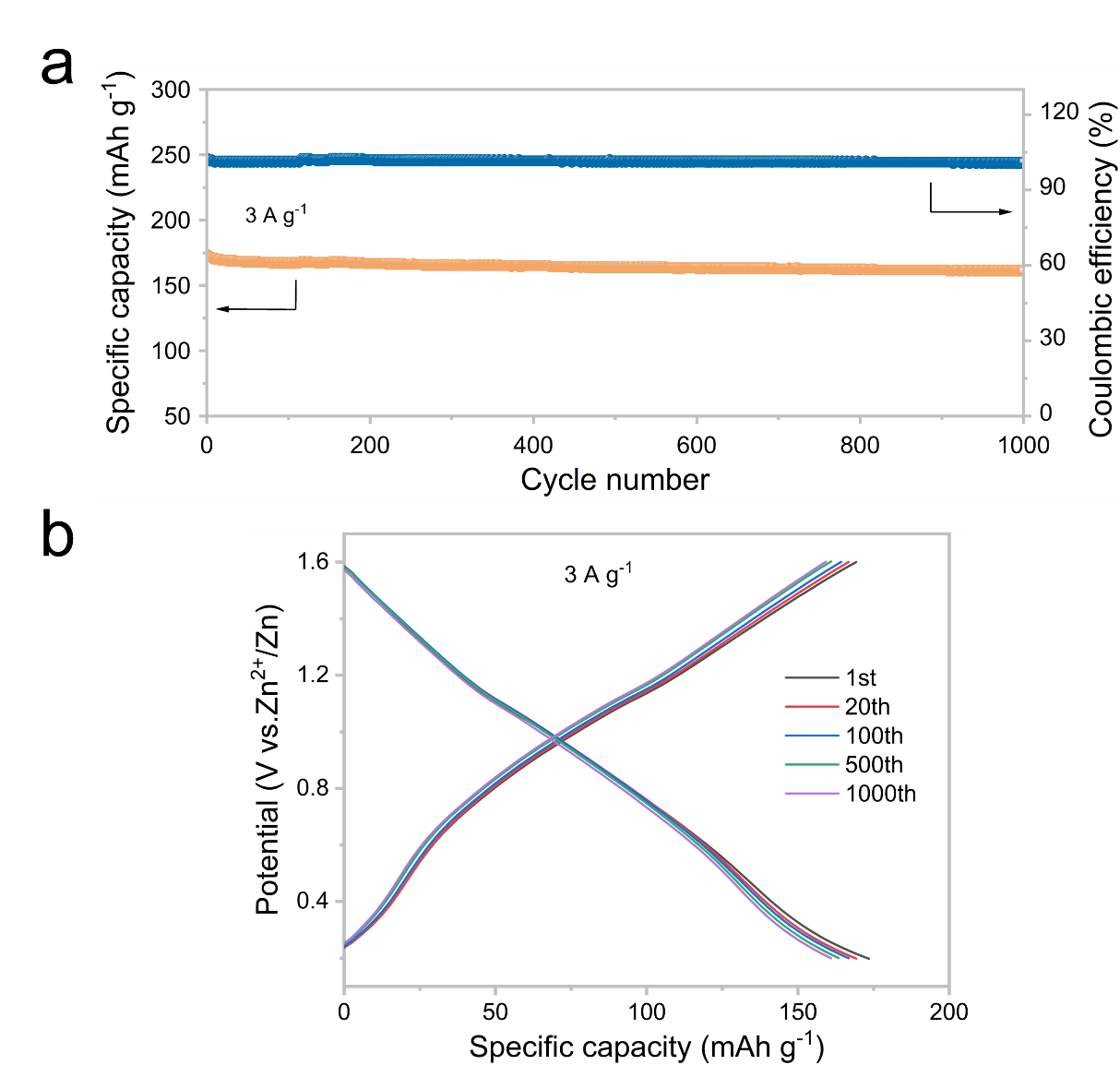


**Figure S31**. (a) Cycling performance of the PTB@MXSC cathode (2.3 mg cm^-2^) at 3 A g^-1^. (b) GCD profiles of the PTB@MXSC cathode (2.3 mg cm^-2^) during cycling at 3 A g^-1^.


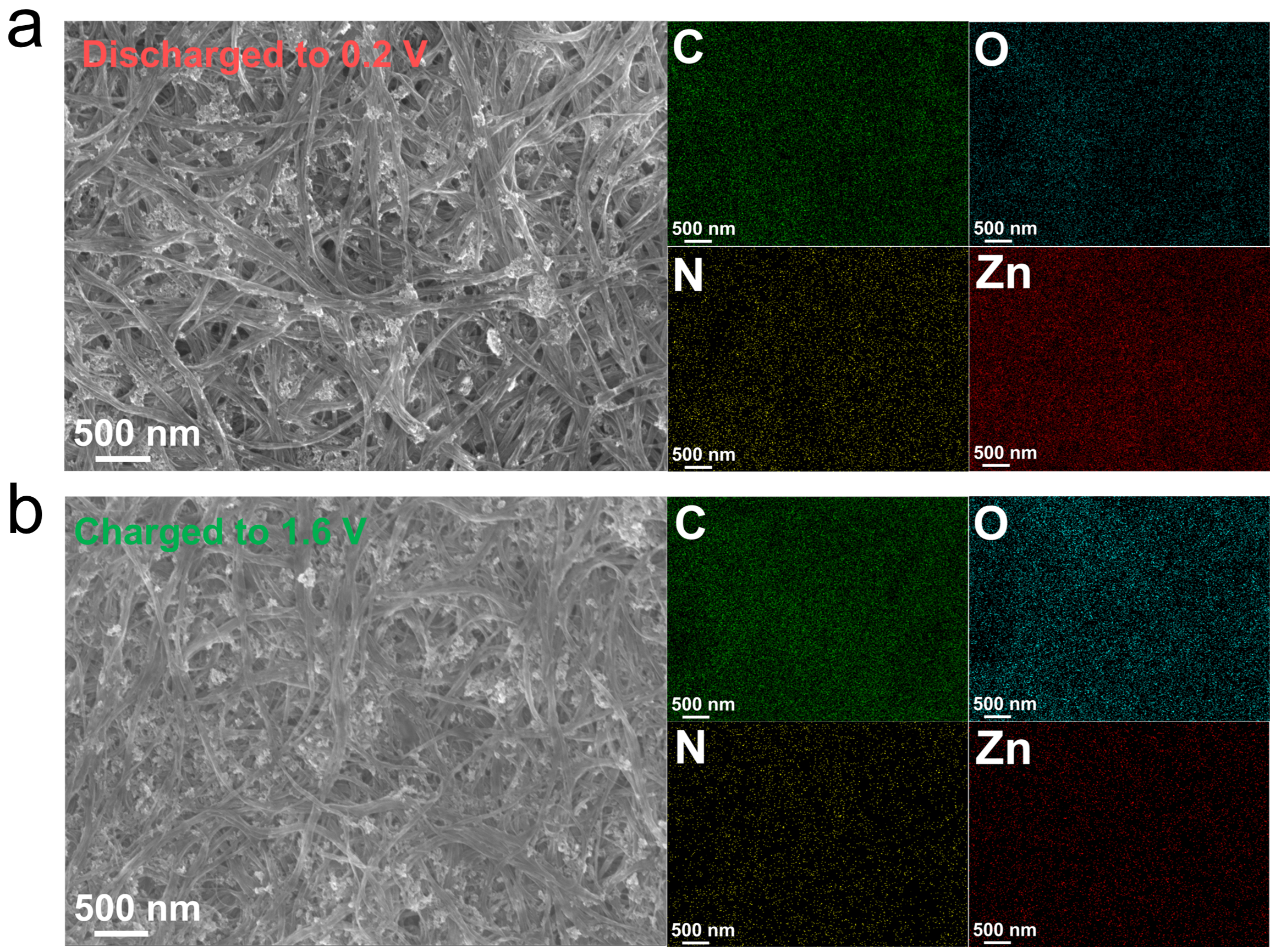


**Figure S32.** SEM and EDS mapping images of PTB@MXSC positive electrode at (a) fully discharged (0.2 V) and (b) fully charged (1.6 V) states.


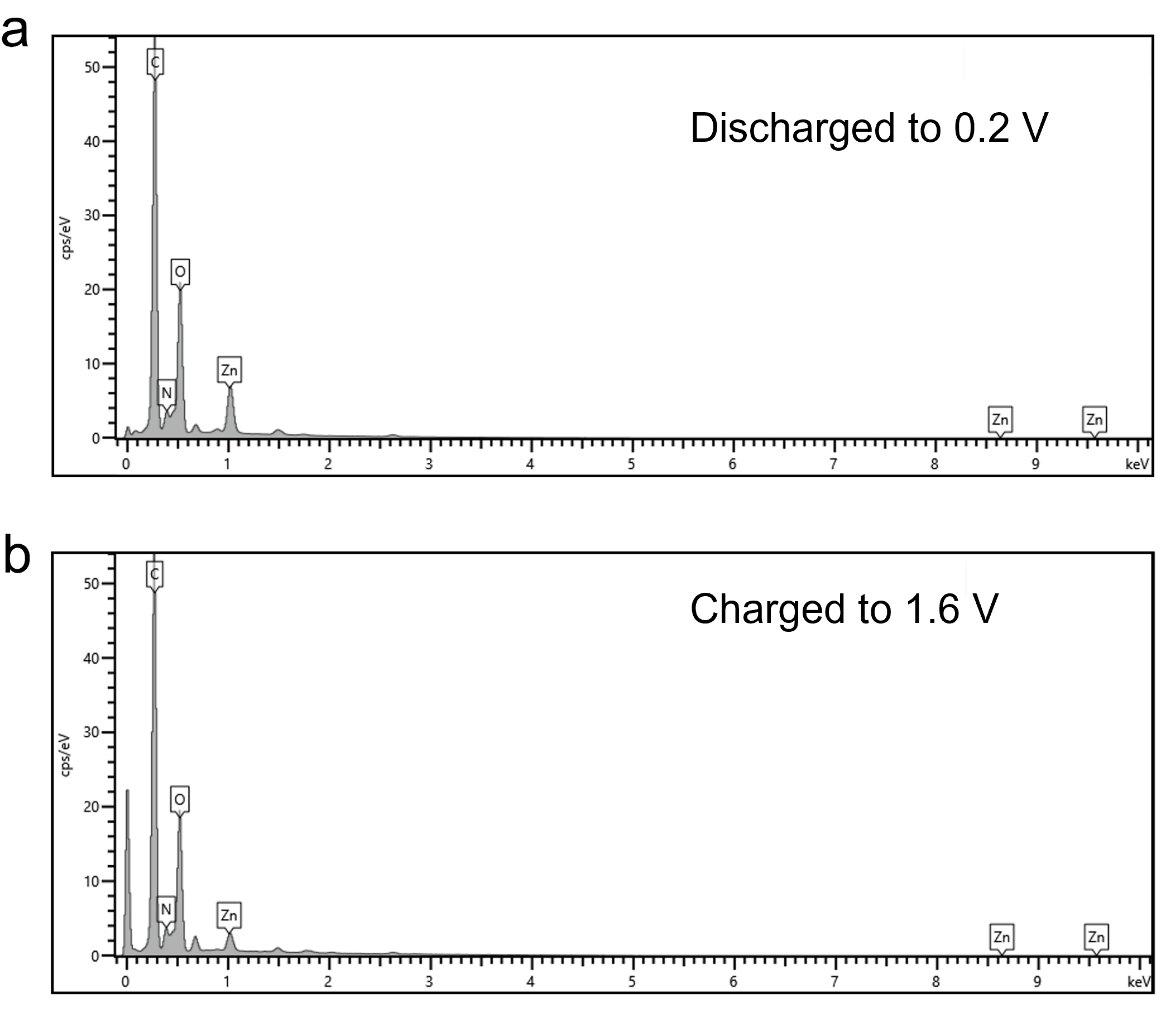


**Figure S33**. Comparative EDS spectra of the PTB@MXSC cathodes at the (a) fully discharged (0.2 V) and (b) fully charged (1.6 V) states.


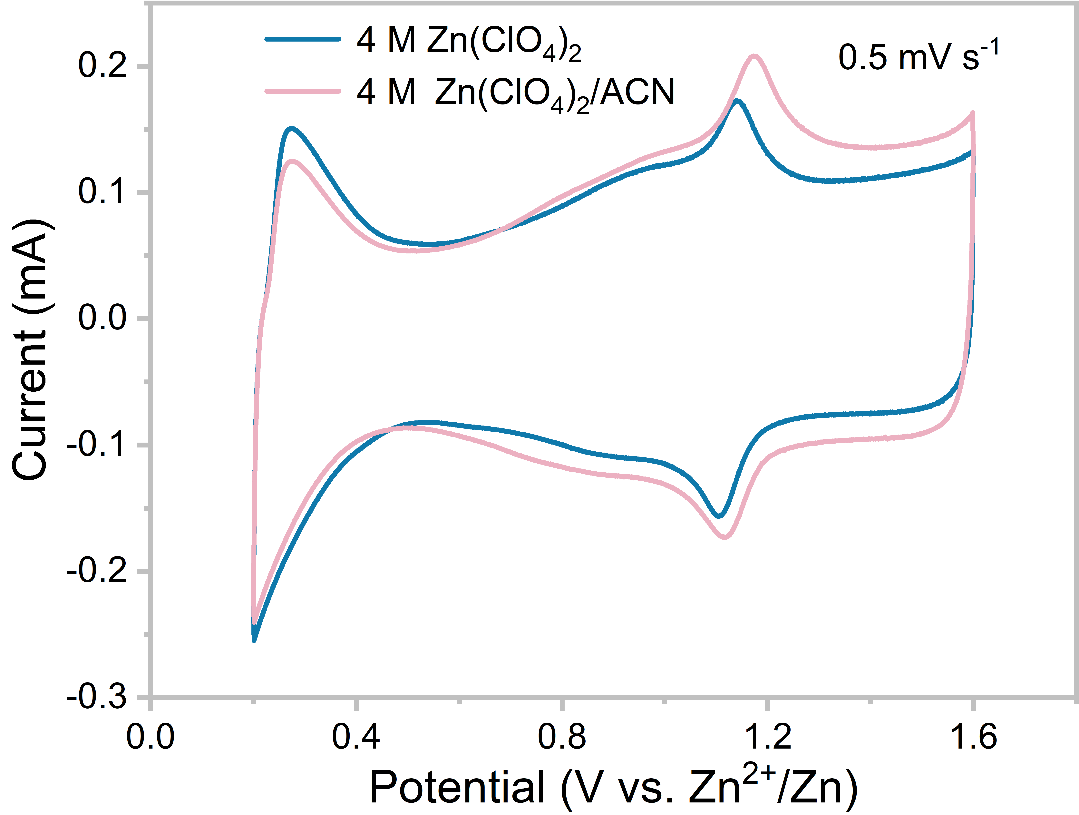


**Figure S34.** CV curves of PTB@MXSC in 4 M Zn(ClO_4_)_2_ and 4 M Zn(ClO_4_)_2_/ACN electrolytes, respectively.


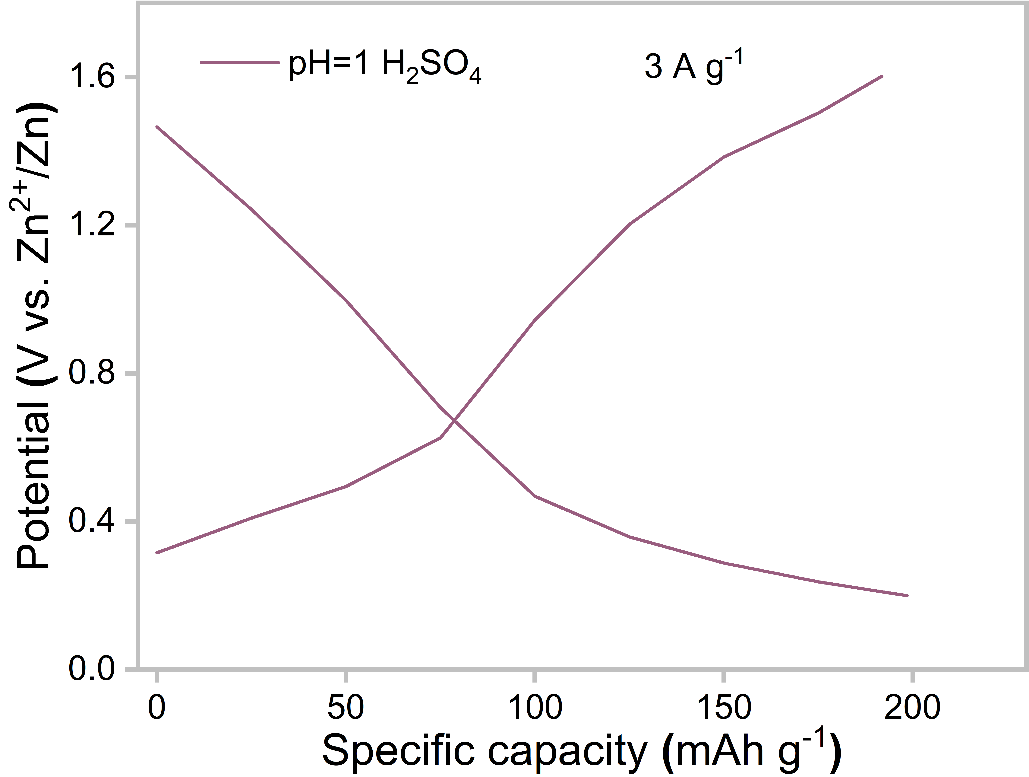


**Figure S35.** GCD curves of PTB@MXSC in pH=1 H_2_SO_4_ electrolyte at 3A g^-1^.


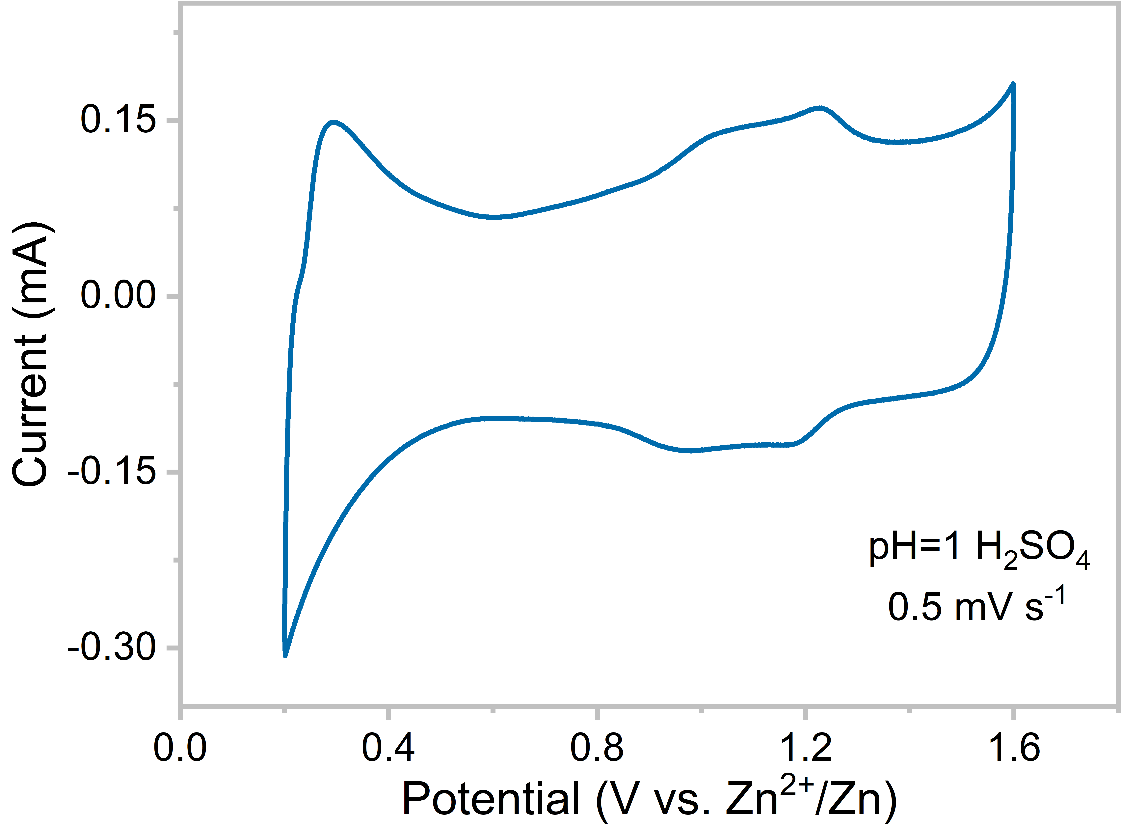


**Figure S36.** CV curve of PTB@MXSC in pH=1 H_2_SO_4_ electrolyte at 0.5 mV s^-1^.


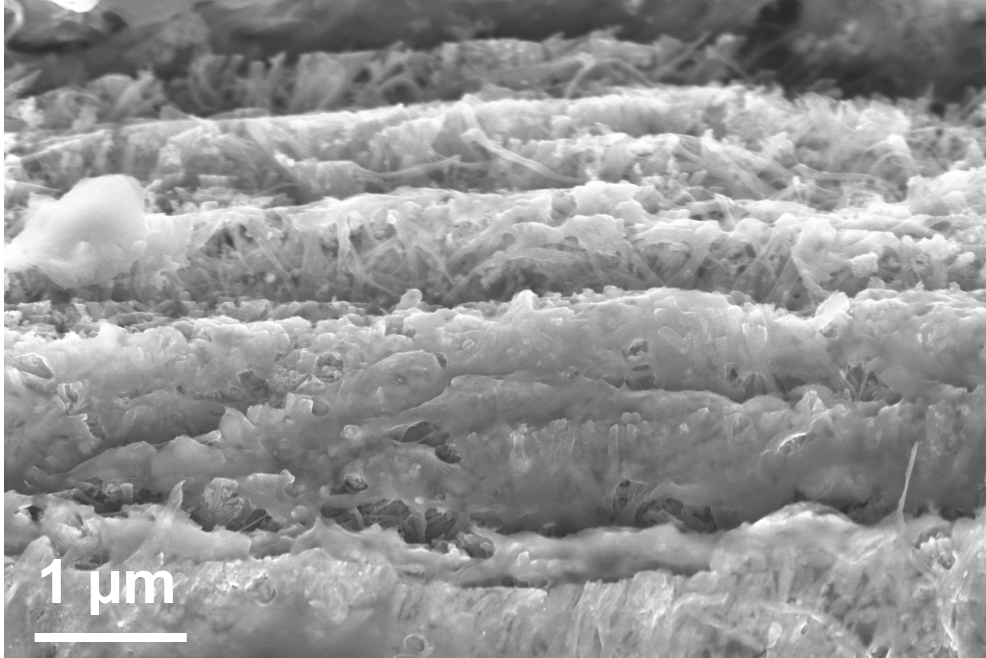


**Figure S37.** Cross-sectional SEM images of the PTB@MXSC after 65,000 cycles at 10 A g^-1^.

**
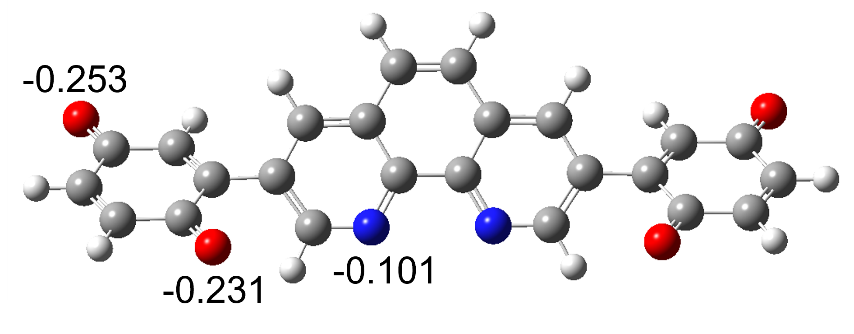
**

**Figure S38.** Mulliken charge values of the target atoms in PTB.


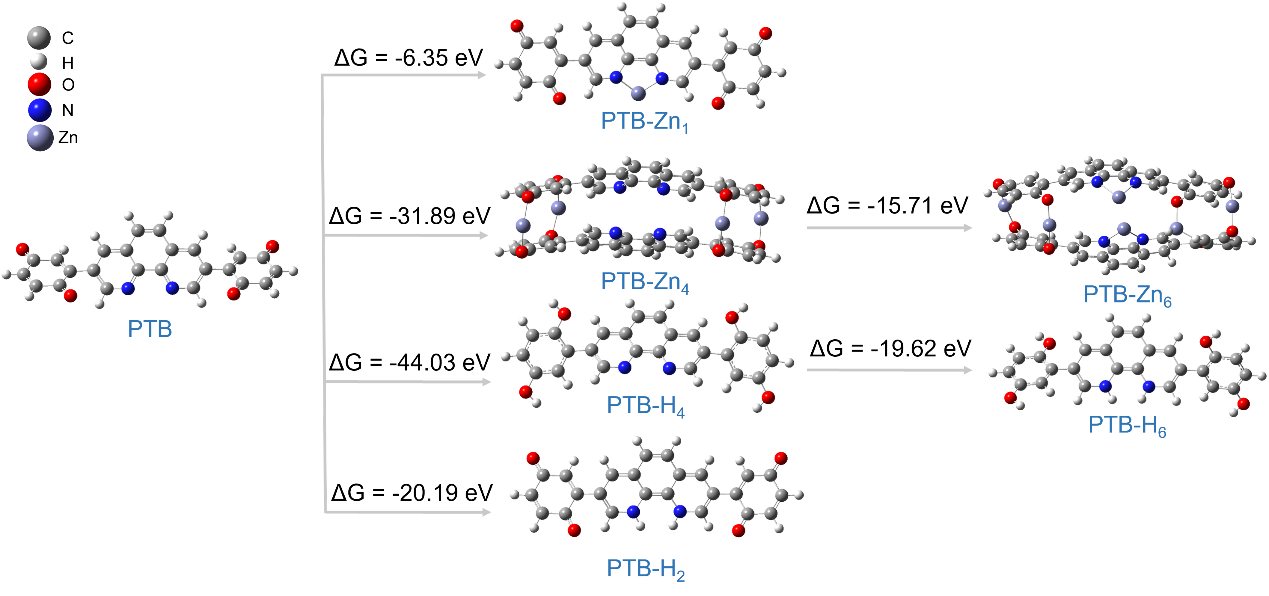


**Figure S39.** Calculated Gibbs free energy of PTB during stepped H^+^/Zn^2+^ coordination process.


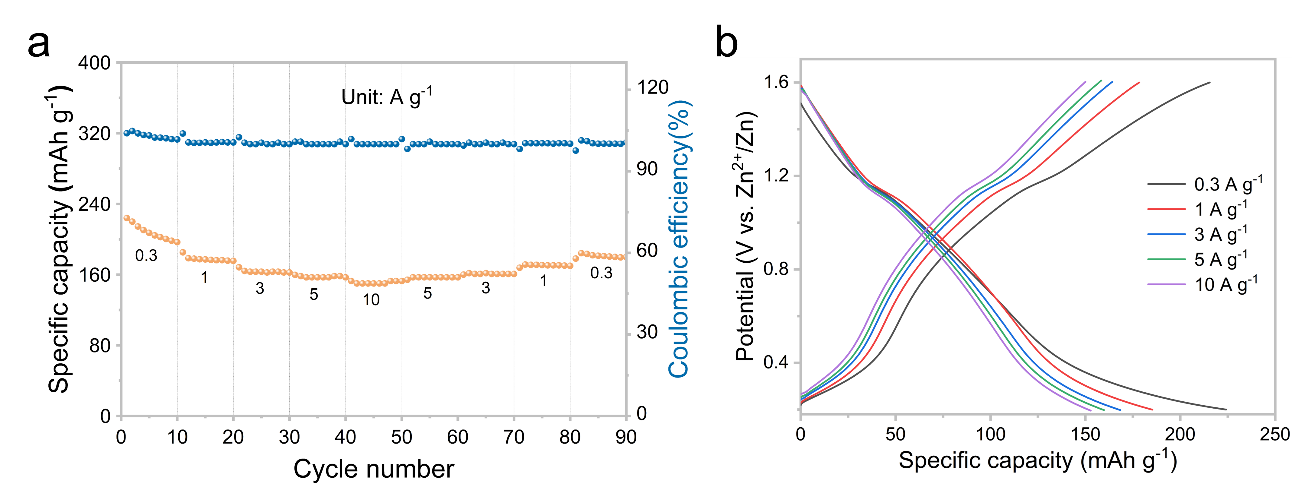


**Figure S40.** (a) Rate performance and (b) GCD profiles at 0.3-10 A g^-1^ of the PTB@MXSC//Zn pouch cell.

**Table S2.** Electrochemical performance comparison of reported organic positive electrode materials for aqueous zinc batteries.

| Organic positive electrode | Cycling performance | Diffusion coefficient (D_Zn_^2+^) (cm^2^ s^-1^) | Reference |
| --- | --- | --- | --- |
| PTB@MXSC | 87.5% after 65000 cycles at 10 A g^-1^ | 10^-8^~10^-7^ | **This work** |
| HOF-HATN | 88% after 10000 cycles at 5 A g^-1^ | 10^-10^~10^-8^ | ^14^ |
| GDAQ | 90.33% after 20000 cycles at 10 A g^-1^ | 10^-12^~10^-8^ | ^17^ |
| PTFHQ-A | 92% after 3400 cycles at 20 A g^-1^ | 10^-11^~10^-10^ | ^18^ |
| TAP/Ti_3_C_2_T_x_ | 81.6% after 10000 cycles at 1 A g^-1^ | 10^-10^~10^-8^ | ^10^ |
| HATN-3CN | 90.7% after 5800 cycles at 5 A g^-1^ | 10^-11^~10^-8^ | ^8^ |
| TA-PTO | 88% after 5000 cycles at 10 A g^-1^ | 10^-11^~10^-9^ | ^19^ |
| HATNQ | 74.8% after 11000 cycles at 5 A g^-1^ | 10^-10^~10^-9^ | ^20^ |
| TABQ-PQ | 90.8% after 30000 cycles at 5 A g^-1^ | 10^-9^~10^-7^ | ^21^ |

**Table S3**. Quantitative EDS Analysis of the PTB@MXSC Cathodes at Fully Discharged and Charged States.

| Discharged to 0.2 V | | Charged to 1.6 V | |
| --- | --- | --- | --- |
| Element | Wt% | Element | Wt% |
| C | 63.64 | C | 68.13 |
| N | 1.90 | N | 2.92 |
| O | 20.05 | O | 22.49 |
| Zn | 14.41 | Zn | 6.47 |
| Total | 100.00 | Total | 100.00 |

**References**

1. J. Yang, P. Xiong, Y. Shi, P. Sun, Z. Wang, Z. Chen and Y. Xu, *Adv. Funct. Mater.*, 2020, **30**(15), 1909597.
2. Z. Zeng, J. Shen, Y. Lai, G. Hua, Y. Wang, J. Liu and W. Tang, *Adv. Funct. Mater.*, 2024, **34**(36), 2401059.
3. A. D. Becke, *J. Chem. Phys.*, 1993, **98**(2), 1372-1377.
4. M. J. Frisch, G. W. Trucks, H. B. Schlegel, G. E. Scuseria, M. A. Robb, J. R. Cheeseman, G. Scalmani, V. Barone, G. A. Petersson, H. Nakatsuji, X. Li, M. Caricato, A. V. Marenich, J. Bloino, B. G. Janesko, R. Gomperts, B. Mennucci, H. P. Hratchian, J. V. Ortiz, A. F. Izmaylov, J. L. Sonnenberg, Williams, F. Ding, F. Lipparini, F. Egidi, J. Goings, B. Peng, A. Petrone, T. Henderson, D. Ranasinghe, V. G. Zakrzewski, J. Gao, N. Rega, G. Zheng, W. Liang, M. Hada, M. Ehara, K. Toyota, R. Fukuda, J. Hasegawa, M. Ishida, T. Nakajima, Y. Honda, O. Kitao, H. Nakai, T. Vreven, K. Throssell, J. A. Montgomery Jr., J. E. Peralta, F. Ogliaro, M. J. Bearpark, J. J. Heyd, E. N. Brothers, K. N. Kudin, V. N. Staroverov, T. A. Keith, R. Kobayashi, J. Normand, K. Raghavachari, A. P. Rendell, J. C. Burant, S. S. Iyengar, J. Tomasi, M. Cossi, J. M. Millam, M. Klene, C. Adamo, R. Cammi, J. W. Ochterski, R. L. Martin, K. Morokuma, O. Farkas, J. B. Foresman, D. J. Fox, Wallingford, CT, 2016.
5. F. Weigend and R. Ahlrichs, *Phys. Chem. Chem. Phys.*, 2005, **7**(18), 3297-3305.
6. W. Humphrey, A. Dalke and K. Schulten, *J. Mol. Graphics.*, 1996, **14**(1), 33-38.
7. T. Lu and F. Chen, *J. Comput. Chem.*, 2012, **33**(5), **33**, 580-592.
8. Z. Ye, S. Xie, Z. Cao, L. Wang, D. Xu, H. Zhang, J. Matz, P. Dong, H. Fang, J. Shen and M. Ye, *Energy Storage Mater.*, 2021, **37**, 378-386.
9. W. Wang, V. S. Kale, Z. Cao, Y. Lei, S. Kandambeth, G. Zou, Y. Zhu, E. Abouhamad, O. Shekhah, L. Cavallo, M. Eddaoudi and H. N. Alshareef, *Adv. Mater.*, 2021, **33**(39), 2103617.
10. X. Wang, Y. Liu, Z. Wei, J. Hong, H. Liang, M. Song, Y. Zhou and X. Huang, *Adv. Mater.*, 2022, **34**(50), 2206812.
11. Y. Wang, H. Cui, R. Li, C. Yue, H. Pan, Z. Tang, X. Wang, Y. Lin, H. Li, C. Han, D. Nan, C. Zhi and H. Lv, *Energy Storage Mater.*, 2024, **65**, 103102.
12. T. Xu, L. Su, C. Ku, Y. Zhang, L. Chen, Q. Gou, S. Fang, P. Xue, M. Tang, C. Wang and Z. Wang, *Chem. Eng. J.*, 2024, **502**, 158169.
13. L. Zhong, C. Wang, J. He, Z. lin, X. Yang, R. Li, S. Zhan, L. Zhao, D. Wu, H. Chen, Z. Tang, C. Zhi and H. Lv, *Adv. Mater.*, 2024, **36**(27), 2314050.
14. J. Chu, Z. Liu, J. Yu, L. Cheng, H.-G. Wang, F. Cui and G. Zhu, *Angew. Chem., Int. Ed.*, 2024, **63**(3), e202314411.
15. Y. Yan, P. Li, Y. Wang, L. Bi, T. W. Lau, M. Miao, S. Yang, Q. Xiong, F. R. Lin, H.-L. Yip, J. Yin, C. Zhi and A. K. Y. Jen, *Adv. Funct. Mater.*, 2025, **35**(21), 2312332.
16. Q. Zhao, W. Huang, Z. Luo, L. Liu, Y. Lu, Y. Li, L. Li, J. Hu, H. Ma and J. Chen, *Sci. Adv.*, 2018, **4**(3), eaao1761.
17. P. Yi, Z. Li, L. Ma, B. Feng, Z. Liu, Y. Liu, W. Lu, S. Cao, H. Fang, M. Ye and J. Shen, *Adv. Mater.*, 2024, **36**(52), 2414379.
18. Q.-Q. Sun, T. Sun, J.-Y. Du, Z.-L. Xie, D.-Y. Yang, G. Huang, H.-M. Xie and X.-B. Zhang, *Angew. Chem., Int. Ed.*, 2023, **62**(35), e202307365.
19. Y. Liu, J. Yu, X. Zhang, D. Han, J. Bai and H.-G. Wang, *Energy Storage Mater.*, 2025, **79**, 104302.
20. Y. Chen, J. Li, Q. Zhu, K. Fan, Y. Cao, G. Zhang, C. Zhang, Y. Gao, J. Zou, T. Zhai and C. Wang, *Angew. Chem., Int. Ed.*, 2022, **61**(37), e202116289.
21. D. Du, J. Zhou, Z. Yin, G. Feng, W. Ji, H. Huang and S. Pang, *Adv. Energy Mater.*, 2024, **14**(21), 2400580.
